# Comparative myoanatomy of *Echinoderes* (Kinorhyncha): a comprehensive investigation by CLSM and 3D reconstruction

**DOI:** 10.1186/1742-9994-11-31

**Published:** 2014-04-05

**Authors:** María Herranz, Michael J Boyle, Fernando Pardos, Ricardo C Neves

**Affiliations:** 1 Dpto. Zoología y Antropología Física (Zoología de Invertebrados), Facultad de Biología, Universidad Complutense de Madrid, C/José Antonio Novais, 2, Madrid 28040, Spain; 2 Smithsonian Tropical Research Institute (STRI), Naos Marine Laboratories, Panama 0843/03092, Republic of Panama; 3 Biozentrum, University of Basel, Klingelbergstrasse 50, CH-4056 Basel, Switzerland

**Keywords:** Ecdysozoa, Scalidophora, Segmentation, Phalloidin, Organ system, Circular muscle, Introvert, Pharynx

## Abstract

**Introduction:**

Kinorhyncha is a clade of marine invertebrate meiofauna. Their body plan includes a retractable introvert bearing rings of cuticular spines, and a limbless trunk with distinct segmentation of nervous, muscular and epidermal organ systems. As derived members within the basal branch of Ecdysozoa, kinorhynchs may provide an important example of convergence on the evolution of segmentation within one of three bilaterian superclades. We describe the myoanatomy of *Echinoderes*, the most specious kinorhynch genus, and build upon historical studies of kinorhynch ultrastructure and gross morphology. This is the first multi-species comparison of a complete organ system by confocal microscopy and three-dimensional reconstruction within Kinorhyncha.

**Results:**

Myoanatomy of adult *Echinoderes* is composed of the following: Head with two mouth cone circular muscles, nine pairs of oral style muscles, ten introvert retractors, one introvert circular muscle, and fourteen introvert circular muscle retractors; Neck with one circular muscle; Trunk showing distinct pairs of ventral and dorsal muscles within segments 1–10, dorsoventral muscles within segments 3–10, diagonal muscles within segments 1–8, longitudinal fibers spanning segments 1–9, three pairs of terminal spine muscles, and one pair of male penile spine muscles; Gut showing a pharynx with ten alternating rings of radial and circular muscle fibers enclosed in a complex sheath of protractors and retractors, an orthogonal grid of longitudinal and circular fibers surrounding the intestine, and paired hindgut dilators.

**Conclusions:**

Myoanatomy is highly conserved between species of *Echinoderes*. Interspecific variation is observed in the arrangement and number of introvert fibers and the composition of pharyngeal muscles. Segmented trunk musculature facilitates the movements of articulated cuticular plates along the anterior-posterior axis. Intersegmental muscle fibers assist with dorsoventral and lateral trunk movements. Protractors, retractors and circular muscles coordinate eversion and retraction of the introvert and mouth cone, and relocation of the pharynx during locomotion and feeding behaviors. Pairs of posterior fibers suggest independent movements of terminal spines, and male penile spines. Within Scalidophora, myoanatomy is more similar between Kinorhyncha and Loricifera, than either group is to Priapulida. Kinorhynch myoanatomy may reflect a convergent transition from vermiform to segmented body plans during the early radiation of Ecdysozoa.

## Introduction

Kinorhyncha is a clade of invertebrate meiofauna that inhabit marine sediments from the intertidal zone to abyssal depths in all major ocean basins [1]. They have a distinctly segmented body plan, with a retractable introvert and a diverse array of cuticular structures along its length. Very little is known about their embryonic development [2], there are no larval life history stages, and only a few studies have examined growth and morphogenesis in pre-adult stages [3-6]. The origin of Kinorhyncha and the relationships within the clade are unresolved. Within Metazoa, kinorhynchs are members of the ‘moulting animals’ known as Ecdysozoa [7], one of two protostome superclades [8-11]. Together, kinorhynchs, priapulids, and most likely loriciferans, form a clade known as Scalidophora, which is the most basal branch in the Ecdysozoa ([12-17], but see [13]). In turn, the scalidophorans share several molecular and morphological similarities with two groups of soft-bodied worms (nematodes, nematomorphs), particularly in the architecture of the brain, which has been a consideration for uniting these five taxa as the more inclusive Cycloneuralia [18]. However, monophyly has not been established for this group [10,11,16,17,19], and therefore the assignment of particular synapomorphic characters within cycloneuralian organ systems, such as digestive, muscular, and nervous tissues, must also remain tentative [15,20,21]. Yet, the kinorhynchs may be the most derived taxon within Scalidophora, or even among the cycloneuralians, since they are the only animals with clearly segmented internal and external structures, including the cuticle, paired and unpaired spines, gland cells, sensory spots, muscles and neurons that collectively represent developmental products of mesodermal and ectodermal tissues along the anterior-posterior axis [14,22,23]. Most importantly, because of their relative position within Ecdysozoa, as a separate and distinct lineage from Panarthropoda, the segmented body plan of kinorhynchs may represent a fascinating example of convergent evolution, and an independent contrast of both the development and function of segmentation not only within Ecdysozoa, but also among other metazoans where segmentation is recognized.

The trunk region of adult kinorhynchs consists of eleven well-defined segments, reflecting an anterior-posterior pattern of repeated elements in the epidermis, nervous system and musculature. Most notable are eleven sets of articulating exoskeletal plates, a ganglionated ventral nerve cord with perpendicular branching of neurites to the peripheral nervous system, and paired sets of functionally specific muscle bands along the trunk [1,24]. Unlike most bilaterians, especially soft-bodied invertebrates, kinorhynchs do not develop the typical set of outer circular and inner longitudinal muscle groups below the epidermis, which must correlate with the production of hard cuticle in their segmented exoskeleton [20]. The continuous circular and longitudinal muscle layers of the body wall have apparently been replaced by isolated muscles attached to the exoskeleton through an intermediate epidermal cell [1,20,25]. Moreover, kinorhynchs do not have limb-like appendages along the body, unlike all of the segmented taxa of Panarthropoda [26]. Amongst others, the absence of such locomotory appendages in kinorhynchs is evidence of a convergent transition from vermiform to segmented body plans that are visible in Panarthropoda and Kinorhyncha, but are missing among all other cycloneuralians. Thus, kinorhynchs are pivotal for understanding the evolution of segmentation. Important similarities and differences in muscle organization between kinorhynchs and arthropods suggest there is still a lot of work to do [5]. Studies of cell lineage, and at least gene expression during segmentation, at analogous sites of appendage development, and within many other organ systems along the kinorhynch body do not exist, but are certainly needed. Until then, it is not possible to infer homology of muscles, nerves or even segments between kinorhynchs and other ecdysozoans with any certainty, at least not beyond predictions based solely on cladistics [5,11,26-28].

Apart from segmentation, kinorhynchs share several notable characteristics with their scalidophoran relatives, including a retractable introvert with rings of chitinous scalids, and the complex myoanatomy that operates it. Within kinorhynch muscle cells, the arrangements of the myofibrils are cross-striated. The exception is an orthogonal grid-like pattern of minute smooth fibers encircling the kinorhynch intestine [5,25,29,30]. Cross-striated myofibrils, although not exclusive to the clade, are thought to facilitate rapid contractions of muscle fibers. As scalidophorans live among the sand grains of marine sediments, and do not have appendages (excluding the toes of the loriciferan Higgins larvae) or locomotory cilia [20,21,31], they must rely on introvert musculature for locomotion and feeding [32-35]. The introvert also thought to have important sensory roles in that environment [1,12,33,36-38]. Furthermore, in each taxon, there are well-developed retractors, protractors, circular muscles, pharynges, and an assortment of external chitinous spines, teeth or scalids [1,21,33,36]. Variation in character states for these and other features are not subtle, and appear to correspond with size, shape, behavior and the presence versus absence of a hard exterior cuticle [1,20,39,40]. Overall, similarities and differences in the function and presumed evolution of characters among kinorhynchs, within Scalidophora, and across Cycloneuralia, are not well understood. Yet, almost all of the internal anatomy, and many external structures, are thought to be directly associated with contractile cells and their fibers [1,14,20,36,41-43]. Thus, thorough descriptions of myoanatomy are essential.

Zelinka [44] and Remane [45] introduced the earliest information on kinorhynch musculature by light microscopy [44,45], which was followed ~ 60 years later by descriptions of ultrastructure using transmission electron microscopy [1,14,33,36]. More recently, kinorhynch musculature was examined by a combination of cytochemical staining and confocal laser scanning microscopy (CLSM) in the cyclorhagid, *Antygomonas* sp. [29], and homalorhagid, *Pycnophyes kielensis*[5,30]. Although each confocal study was limited in scope, and to a single species, musculature was selectively labeled and examined as an organ system, setting up the first comparisons between kinorhynchs and other ecdysozoans, and metazoans in general. In this manuscript, we present a comprehensive description of the muscular organ system in *Echinoderes*, the largest kinorhynch genus with nearly 80 species and a global distribution. We have selected five species, from both sides of the Atlantic Ocean, that exemplify morphological variability within the genus based upon a range of taxonomic characters: *Echinoderes horni* Higgins, 1983 [46]; *Echinoderes spinifurca* Sørensen et al., 2005 [47]; *Echinoderes dujardinii* Claparède, 1863 [48]; *Echinoderes hispanicus* Pardos Higgins and Benito, [49] and *Echinoderes* sp. (see Table 1). Thus far, the myoanatomy of *Echinoderes* has not been investigated with confocal laser scanning technology. Therefore, we have utilized a comparative multi-species approach, and have rendered the first complete three-dimensional reconstructions of kinorhynch musculature with 3D imaging software. Particular emphasis has been placed on the arrangement and function of muscles within the introvert, pharynx and associated structures at the anterior end of the kinorhynch body plan. Results of our investigation supplement previous studies with new observations and interpretations, and enable a broader discussion of comparative myoanatomy within Kinorhyncha, and between kinorhynchs and closely related groups, the Loricifera, Priapulida and Nematomorpha.

**Table 1:** **Comparative difference in size, shape, and spines in five species of *Echinoderes *** **of which muscle architecture was investigated**

	**Trunk length (μm)**	**Outline**	**Middorsal spines**	**Tergal extensions**
*E. dujardinii*	350	Barrel shaped	5 short	Short
*E. hispanicus*	300	Elongated	3 long	Short
*E. horni*	270	Elongated	None	Short
*E. spinifurca*	270	Elongated	5 long	Long
*Echinoderes* sp*.*	270	Bulbous anterior end	1 short	Short

## Results

### External morphology of *Echinoderes*

**Figure 1:**
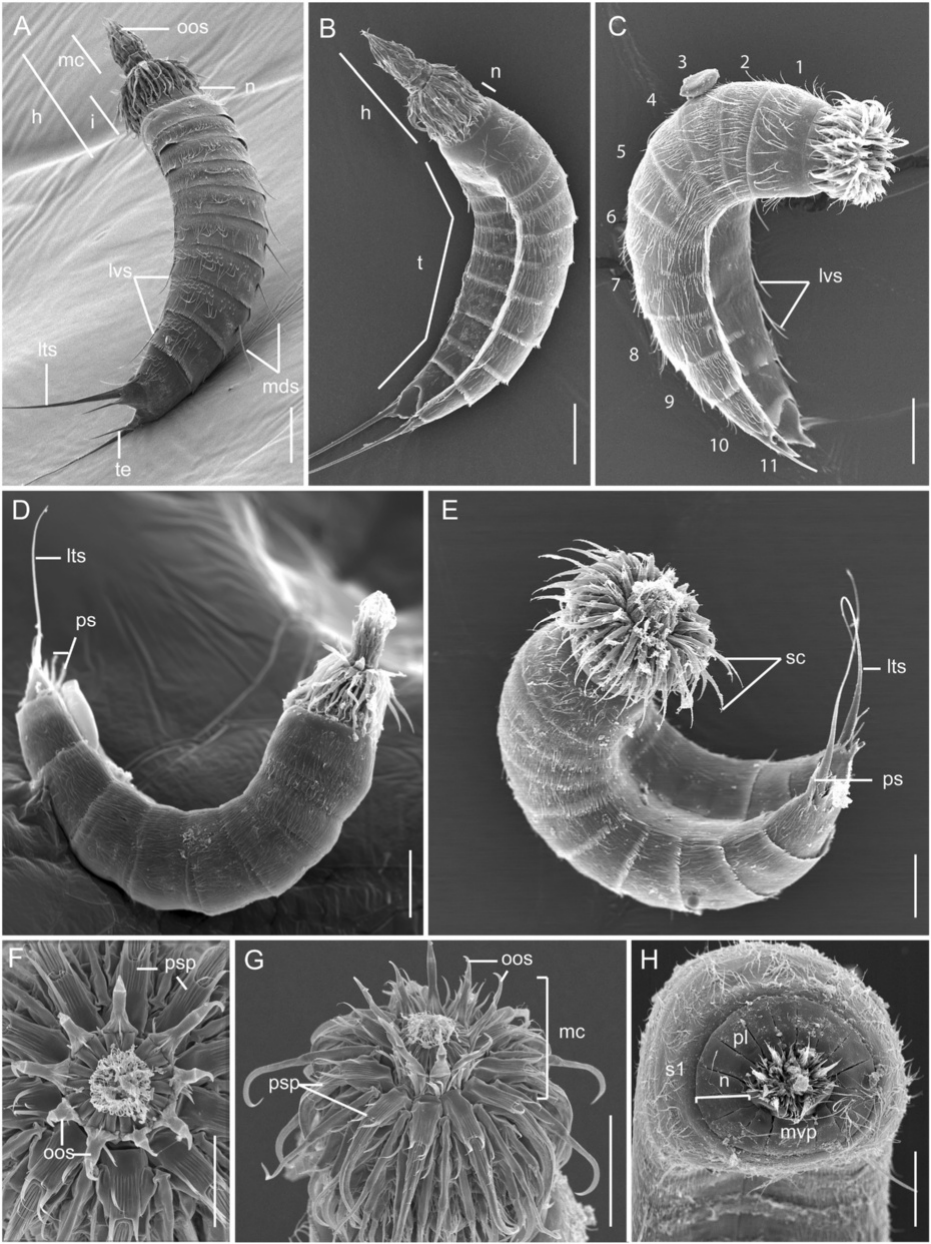
**The external morphology of five species of *Echinoderes *(Kinorhyncha). (A-H)** Scanning Electron Microscopy (SEM). **(A)***Echinoderes spinifurca,* lateral view, with ventral to the left, anterior to the top. Introvert and mouth cone are everted. **(B)***Echinoderes horni,* lateroventral view, anterior to the top. Introvert and mouth cone are everted. **(C)***Echinoderes hispanicus,* lateroventral view, anterior to the right. Introvert is everted, and the mouth cone is retracted. **(D)***Echinoderes* sp., lateral view, with anterior and posterior toward the top, dorsal to the bottom. Introvert and mouth cone are everted. **(E)***Echinoderes dujardinii,* lateroventral view, anterior and posterior toward the top. Introvert is everted and part of mouth cone is visible. **(F)***Echinoderes spinifurca,* apical view of the mouth cone. **(G)***Echinoderes spinifurca,* lateral view of the introvert and mouth cone, anterior to the top. **(H)***Echinoderes dujardinii,* apical view. Introvert and mouth cone are retracted, and placids of the neck are closed. Abbreviations: h, head; i, introvert; lts, lateral terminal spine; lvs, lateroventral spine; mc, mouth cone; mds, middorsal spine; mvp, midventral placid; n, neck; oos outer oral style; pl, placid; ps, penile spine; psp, primary spinoscalid; s, segment; sc, scalids; t, trunk; te, tergal extension. Digits refer to segment number. Scale bar, 30 μm.

**Figure 2:**
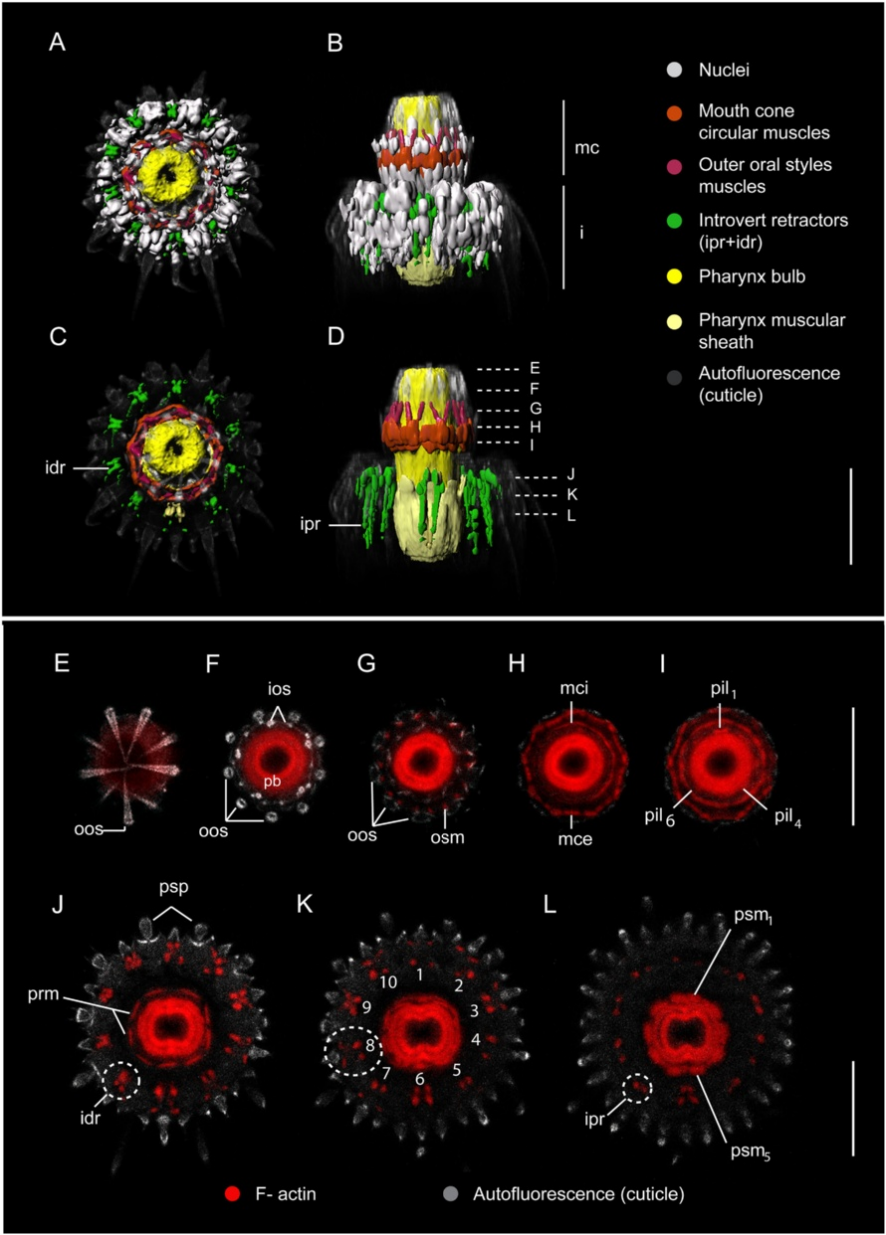
**Myoanatomy of the mouth cone and introvert of *Echinoderes spinifurca. *(A-D)** 3D reconstructions of confocal z-stacks in the head region. **(E-L)** confocal z-stack projections of phalloidin staining in the head region. **(A, C)** apical view, ventral side is down. **(B, D)** ventral view, anterior to the top. Cell nuclei are shown in **A** and **B**, but not shown in **C** and **D** for clarity. **(E-L)** Phalloidin staining in the same specimen as in **A**-**D**. Series of optical cross-sections by depth through the head as indicated by dashed lines in **D**; ventral side is down in all images. **E**-**I**, mouth cone region; **J**-**L**, introvert region. Numbers in **K** refer to the ten introvert retractors. Note: introvert scalids do not contain musculature. Abbreviations: idr, introvert distal retractors; ipr, introvert proximal retractors; ios, internal oral style; mce, mouth cone external circular muscle; mci, mouth cone internal circular muscle; oos, outer oral style; osm, outer oral style muscle; pb, pharynx bulb; pil, pharynx inner longitudinal muscle; prm, pharynx retractor muscle; psm, pharynx sheath muscle; psp, primary spinoscalids. Scale bars, 30 μm.

As observed in all known kinorhynch genera, species of *Echinoderes* exhibit a bilaterally symmetric body plan that is divided into three regions along the anterior-posterior axis: head, neck and trunk (Figure 1A-E). The head region is unsegmented, and is composed of an eversible introvert with a retractable mouth cone. The introvert bears seven concentric rings of cuticular appendages, the scalids, which encircle the mouth cone; the scalids from the first ring are named primary spinoscalids (psp) (Figures 1A-B, G and 2A-F). The mouth cone includes nine articulated outer oral styles (Figure 1F, G), and three rings of inner oral styles that surround a centralized mouth within the terminal end of the cone. The neck region is also unsegmented, and is composed of sixteen cuticular plates, the placids. All of the placids are interconnected by soft cuticle, and they form a closing system for the anterior end of the trunk when the introvert is retracted (Figure 1H). The trunk is divided into eleven articulating segments (Figure 1A-E). The first and second segments are ring-like, however, segments 3–11 are each composed of one tergal (dorsal), and two sternal (ventral) plates. Particular trunk segments may have relatively long, cuticular spines extending from the surface of their plates. Spines typically extend in a posterior direction from a middorsal and/or lateroventral surface, or from the terminal end of segment 11 (Figure 1A-E). Males have three pairs of penile spines (Figure 1D-E), and females possess a single pair of lateral terminal accessory spines. The primary differences in external morphology among species of *Echinoderes* include the size, number, and arrangement of cuticular spines, as well as notable variation in the presence and relative positions of other cuticular structures such as sensory spots, tubes, hairs and glands. Moreover, the length and proportion of trunk regions are highly variable among species.

### Myoanatomy of *Echinoderes*

A comparison among the five species of *Echinoderes* in this study reveals a similar pattern of myoanatomy, regardless of detectable differences in external morphology (Table 1, Figure 1A-E). For clarity, we divide our descriptions of musculature into head, neck, trunk and gut regions of the kinorhynch body plan. When observed, species-specific differences in myoanatomy are noted for a particular region.

#### Musculature of the head: mouth cone

**Figure 3:**
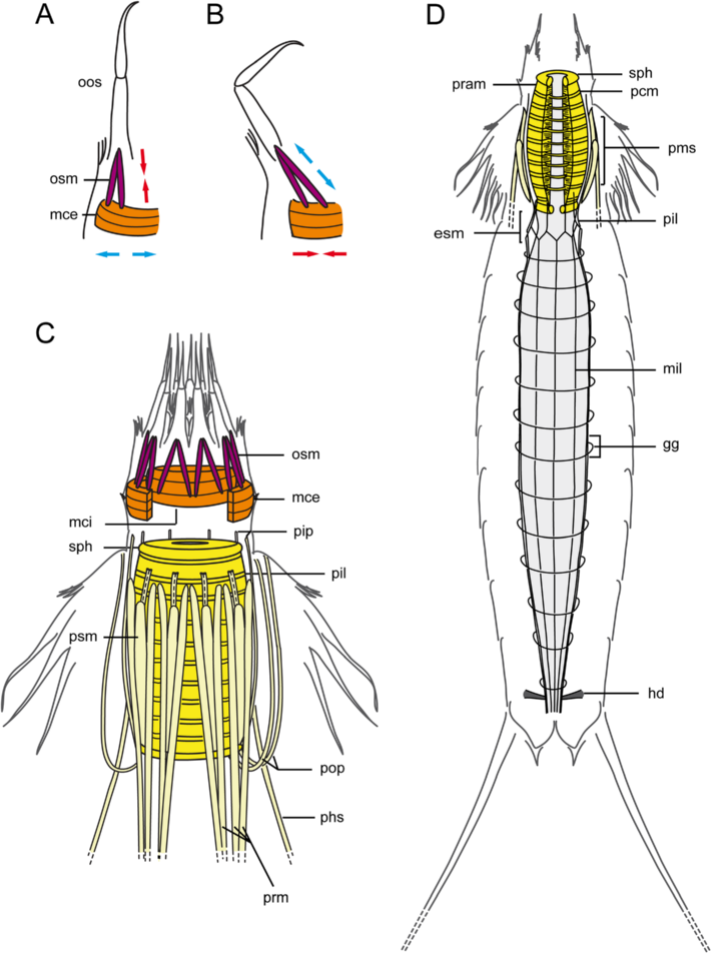
**Schematics of musculature within subregions of the gut in *Echinoderes*. (A-B)** predicted antagonistic functions between muscles associated with the outer oral styles and the mouth cone external circular muscle. Red arrows mean contraction whereas blue arrows extension. **(C)** myoanatomy of the mouth cone and pharynx in lateral view, with ventral side to the right. **(D)** overview of myoanatomy in the pharynx and intestine, in ventral view, with anterior to the top. Color-code according with 3D reconstructions of Figures 2 and 8. The gut is depicted in light grey. Abbreviations: esm, esophagus muscles; gg, gut grid; hd, hindgut dilators; mce, mouth cone external circular muscle; mci, mouth cone internal circular muscle; mil, midgut inner longitudinal muscle; oos, outer oral style; osm, outer oral styles muscles; pcm, pharynx circular muscle fibers; phs, pharynx suspensor; pil, pharynx inner longitudinal muscle; pip, pharynx internal protractor; pms, pharynx muscular sheath; pop, pharynx outer protractor; pram, pharynx radial muscle fibers; prm, pharynx retractor muscle; psm, pharynx sheath muscles; sph, sphincter.

Myoanatomy of the mouth cone includes longitudinal and circular muscle groups. There are eighteen short longitudinal muscles, arranged in nine pairs, situated at the bases of the outer oral styles (oos) (Figure 2G). These are the outer oral styles muscles (osm). One pair (osm) is located at the base of each outer oral style (Figures 2B, D, G and 3A-C) and the two longitudinal muscles of a single pair converge at their anterior end to form an inverted V-shape (Figure 2B, D). One V-shaped pair of muscles (osm) is associated with one outer oral style (oos) (Additional file 1, Figure 3A).

**Figure 4:**
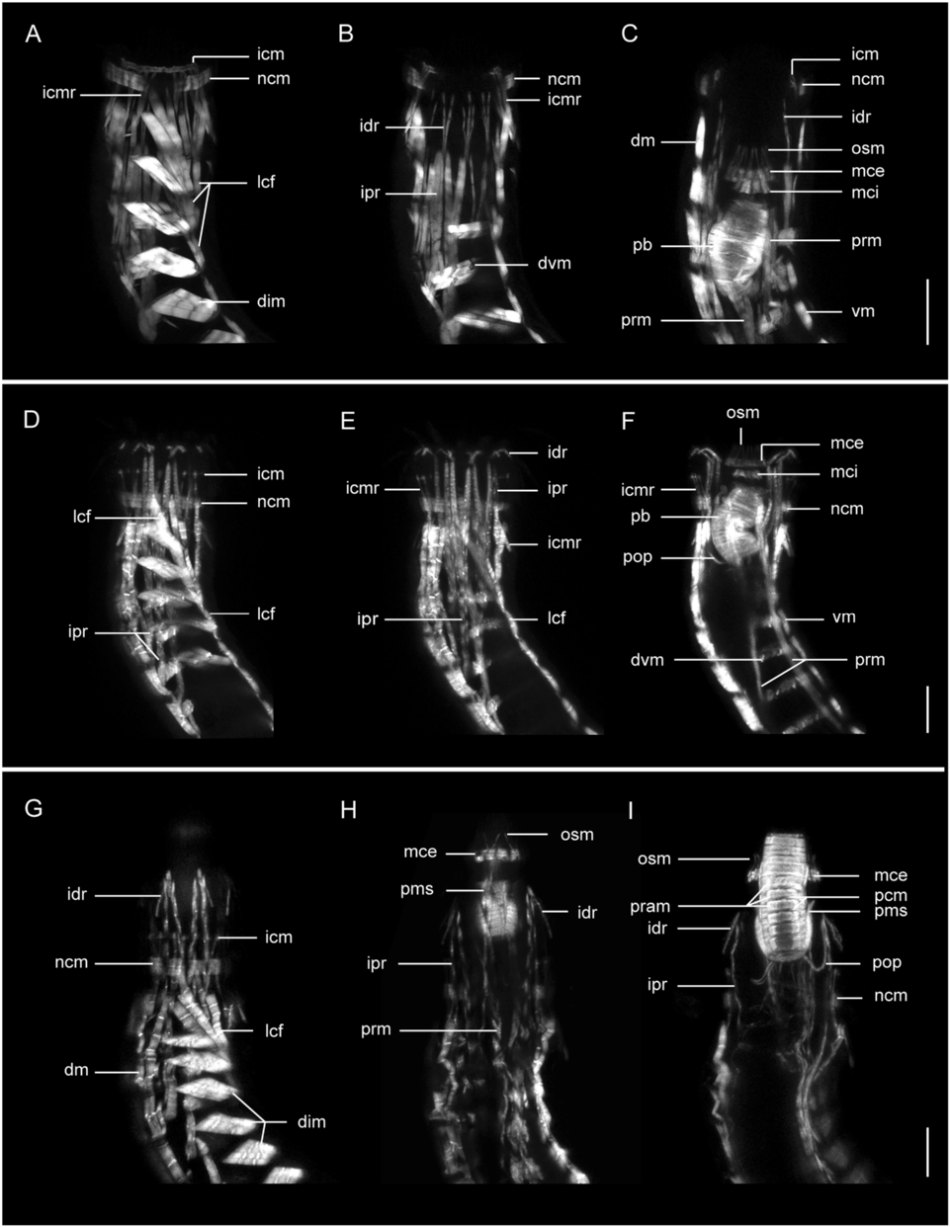
**Musculature of the anterior regions in three species of *Echinoderes.*** Confocal z-stack projections of phalloidin labeling. Three sets of images **(A-C; D-F; G-I)** each show a species-specific series of consecutively deeper z-stacks toward the pharynx. Anterior is to the top in all images. **(A-C)***Echinoderes horni,* head retracted. **(D-F)***Echinoderes hispanicus,* head partially extended. **(G-I)***Echinoderes spinifurca*, head fully extended. Abbreviations: dm, dorsal muscle; dim, diagonal muscle; dvm, dorsoventral muscle; icm, introvert circular muscle; icmr, introvert circular muscle retractor; idr, introvert distal retractor; ipr, introvert proximal retractor; lcf, longitudinal continuous fiber; mce, mouth cone external circular muscle; mci, mouth cone internal circular muscle; ncm, neck circular muscle; osm, outer oral style muscle; pb, pharynx bulb; pcm, pharynx circular muscle fibers; pop, pharynx outer protractor; pms, pharynx muscular sheath; pop, pharynx outer protractor; pram, pharynx radial muscle fibers; prm, pharynx retractor muscle; vm, ventral muscle. Scale bar, 20 μm.

**Figure 5:**
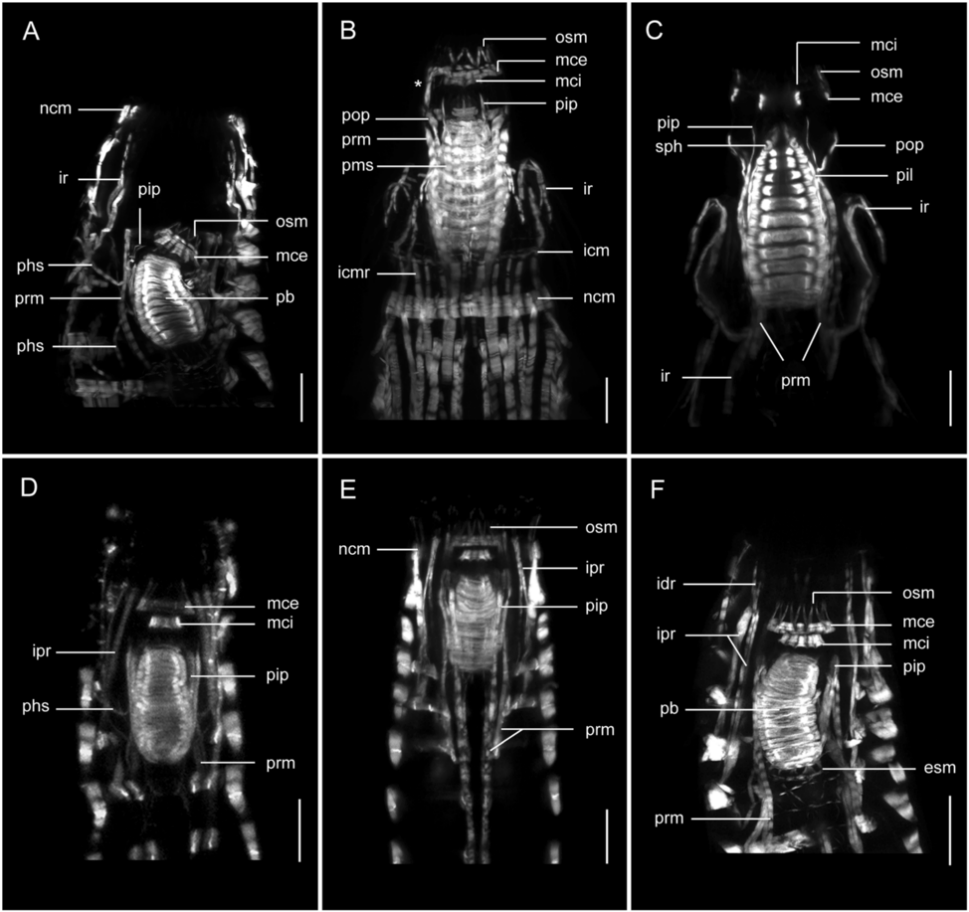
**Musculature of the pharynx, introvert and mouth cone in *Echinoderes*.** Confocal z-stack projections of phalloidin staining within the pharynx at different degrees of retraction. Ventral views, with anterior to the top in all images. **(A-C)***Echinoderes dujardinii*. **(D-F)***Echinoderes spinifurca.* In each micrograph, there are distinct muscle fibers connecting with, and enclosing, the pharyngeal bulb, including protractors, retractors, suspensors, and fibers of the pharynx sheath. An asterisk marks a muscle connecting the pharynx with the mouth cone external circular muscle. Abbreviations: esm, esophagus muscles; icm, introvert circular muscle; icmr, introvert circular muscle retractors; idr, introvert distal retractor; ipr, introvert proximal retractor; ir, introvert retractor; mce, mouth cone external circular muscle; mci, mouth cone internal circular muscle; ncm, neck circular muscle; osm, outer oral style muscle; pb, pharynx bulb; phs, pharynx suspensor; pip, pharynx internal protractor; pms, pharynx muscular sheath; pop, pharynx outer protractor; prm, pharynx retractor muscles; sph, sphincter. Scale bar, 20 μm.

There are two concentric rings of mouth cone circular muscles, one external and one internal. Each muscular ring is composed of three fibers, which are located within the basal part of the mouth cone (Figures 2A-D, H, I, 3C and 4C, F, I). The external circular muscle (mce) is positioned along the bases of the outer oral styles, and oral styles muscles (Figures 2D, H and 3C). The internal circular muscle (mci) is situated basally to the inner oral styles, and likely associated with the pharynx (Additional file 1, Figures 2H-I and 3C). Both external and internal circular muscle rings appear to be attached to the soft cuticle in between the bases of their respective oral styles (Figure 2H-I). This is more noticeable in the external circular muscle, which shows a polygonal shape in cross section (Figure 2H). The three rows of inner oral styles are not associated with a myoanatomical feature other than the mouth cone internal circular muscle described above (Figure 2F). The external and internal circular muscles move independently from each other, and may be observed at different levels along the anterior-posterior axis of the mouth cone (Figures 2D, 4C, F, I and 5A-F).

#### *Musculature of the head*: *introvert*

The myoanatomy of the introvert includes a set of longitudinal introvert retractors, an introvert circular muscle, and a set of longitudinal introvert circular muscle retractors. The introvert retractors are composed of one or two sets of longitudinal muscles. There are ten groups of 2–3 short, thin distal retractor muscles (idr) that probably insert into the anterior part of the introvert (Figures 2C, J, K, 4B-I and 5D-F). These (idr) muscles alternate positions with the primary spinoscalids (psp) and bend toward the bases of the spinoscalids (Additional file 1, Figure 2C-D, J). The spinoscalids themselves do not possess musculature (Additional file 1, Figure 2C-D, J, K).

The distal retractor muscles (idr) are connected with ten pairs of longitudinal proximal retractor muscles (ipr), which extend in an anterior-posterior direction between the pharynx and the trunk musculature (Additional file 1, Figures 2D, L, 4B, E, H and 5D-F). Each muscle in a pair of proximal retractor muscles (ipr) is composed of 2–3 fibers. The posterior ends of the proximal introvert retractors attach dorsolaterally or ventrolaterally, most likely at the segmental pachycycli (cuticular thickening situated at the anterior margin of a segment) from trunk segment 3 to segment 5 (Figures 4B, E, D, G and 5D-F).

In *E. dujardinii*, there is no distinction between proximal and distal retractor muscles. Instead, there are ten pairs of long continuous introvert retractors (ir) composed of four fibers each. These introvert retractors attach posteriorly in the same trunk segments as in the other *Echinoderes* species, and bend anteriorly in a swan-neck-shaped configuration toward the bases of the spinoscalids (Figure 5A-C). Only the two central fibers, out of four fibers, bend toward the spinoscalids. The remaining two short fibers are positioned adjacent to the longer, curved fibers.

**Figure 6:**
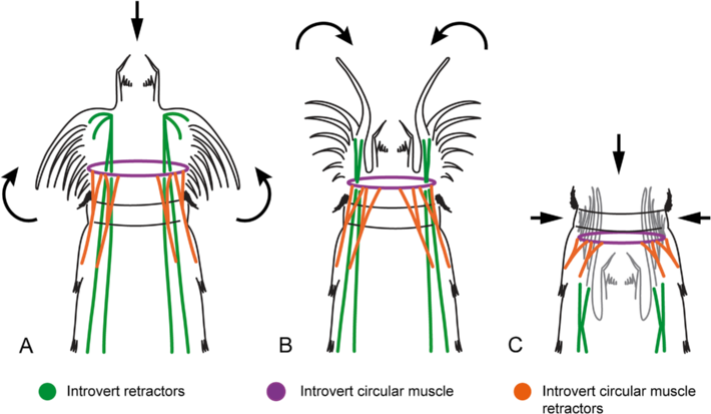
**Schematic of muscle function during introvert retraction in *Echinoderes*.** Transition from full eversion **(A)** to full retraction **(C)** of the introvert. Anterior is to the top. Only the muscle groups considered to be directly involved with introvert retraction are represented. Arrows indicate the directional movements of the introvert and scalids. **(A)** Introvert and mouth cone are everted. Introvert retractors, introvert circular muscle, and introvert circular muscle retractors are stretched. **(B)** Introvert is half-retracted, and the mouth cone is retracted. Introvert distal retractors are contracted, introvert proximal retractors are stretched, introvert circular muscle is contracted to a smaller diameter than the neck, and introvert circular muscle retractors are stretched. **(C)** Introvert and the mouth cone are fully retracted. Introvert retractors, introvert circular muscle, and introvert circular muscle retractors are now fully contracted and located within the trunk.

The introvert circular muscle (icm) is composed of 3–5 thin fibers, and its position changes depending on the position of the introvert (Figure 6). When the introvert is everted, the introvert circular muscle is relaxed and situated at the level of the last row of spinoscalids (Figures 4D-I and 6A). In this position, the diameter of this circular muscle is larger than the diameter of the neck (Figures 4D-F and 6A). However, when the introvert is fully retracted, the introvert circular muscle is constricted to a smaller diameter (Figures 4A-C and 6C) and situated below the level of the neck, within the anterior region of the first trunk segment (Figure 6C). There are approximately 14–16 short longitudinal muscles that are probably attached to the introvert circular muscle (Figure 6); their anterior ends are bifurcated where they meet the introvert circular muscle, and their posterior ends extend toward the anterior region (pachycyclus) of the second trunk segment. These short muscles are the introvert circular muscle retractors (icmr) and extend internally along the region of the neck (Figures 4A, D, E, F, 5B and 6) (see below). When the introvert is everted, these muscles (icmr) are extended to reach their maximum length (Figure 6A). Inversely, when the introvert is retracted, the introvert circular muscle retractors contract to their minimum length (Figure 6C).

#### Musculature of the neck

**Figure 7:**
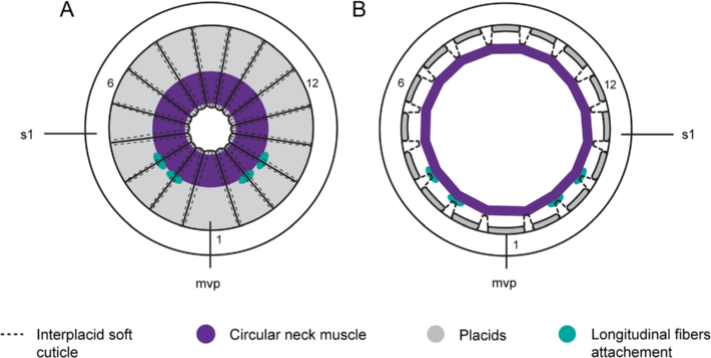
**Schematic of the neck circular muscle and closing system in *Echinoderes*.** Apical views, with ventral side to the bottom. **(A)** Neck circular muscle is contracted, and placids are rotated down toward the center of the neck; the neck is closed. **(B)** Neck circular muscle is stretched along interplacid attachment sites (dashed lines), and the placids are ‘flipped open’. The neck is open. Note the attachments of longitudinal fibers from the trunk to interplacid soft cuticle. Abbreviations: mvp, midventral placid; s1, segment 1.Numbers refer to the placid numeration starting with the midventral placid clockwise.

**Figure 8:**
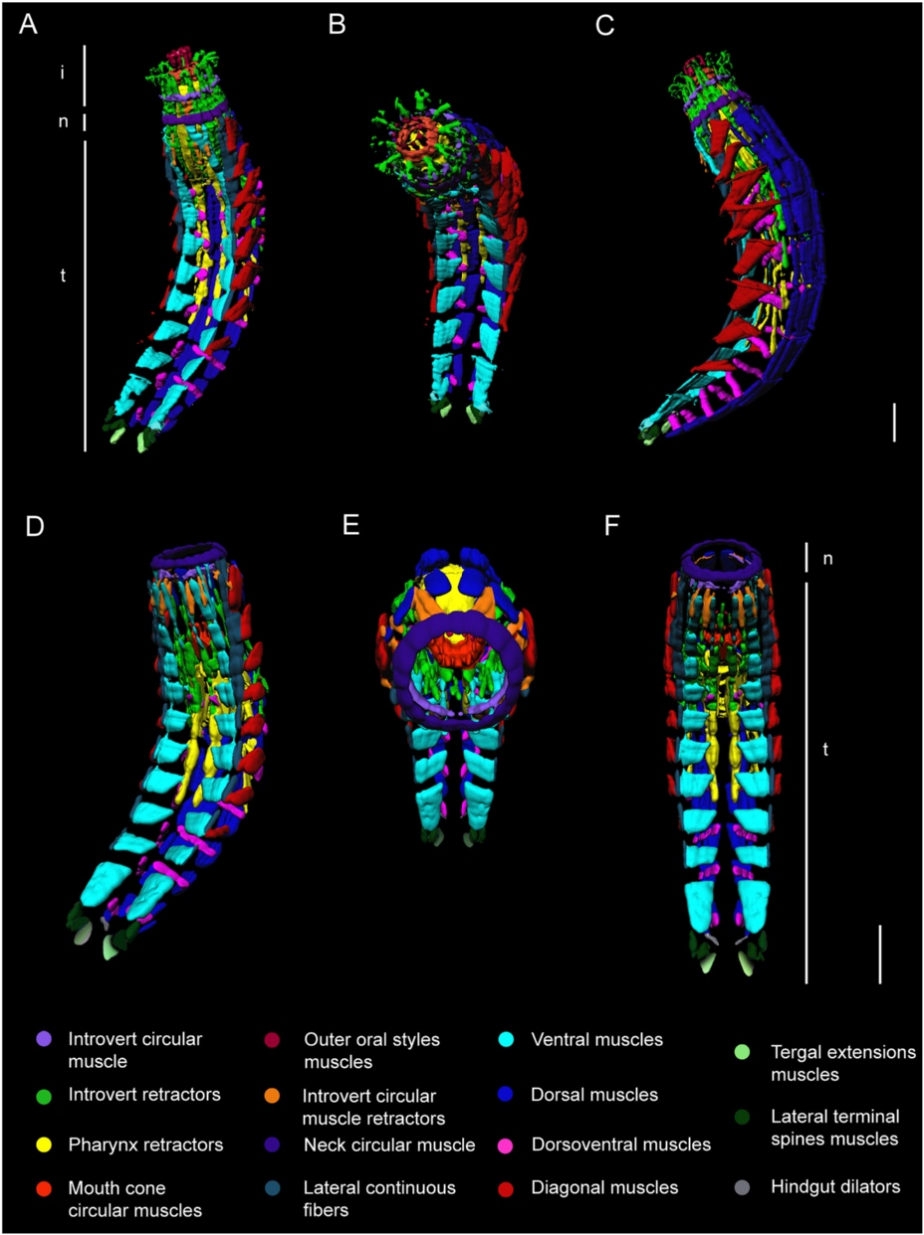
**Three-dimensional reconstruction of myoanatomy in *Echinoderes horni*. (A-C)** Single specimen with the introvert fully everted. **(D-F)** Single specimen with the introvert fully retracted. Anterior to the top in all images. Notable musculature: **(A)** lateroventral view: outer oral style muscles, neck circular muscle, segmented trunk muscles; **(B)** lateroventral, with introvert in apical view: mouth cone circular muscles, introvert retractors; **(C)** lateral view: diagonal muscles, dorsal muscles, dorsoventral muscles; **(D)** lateroventral view: ventral muscles, lateral continuous fibers, terminal spine muscles; **(E)** apical-ventral view showing introvert and mouth cone within the trunk: neck circular muscle, introvert circular muscle; **(F)** ventral view: introvert circular muscle retractors, ventral muscles, pharynx retractors, terminal spine muscles, tergal extension muscles, hindgut dilators. Abbreviations: i, introvert; n, neck; t, trunk. Scale bar, 30 μm.

The neck region contains a circular muscle (ncm) composed of three wide fibers. This neck circular muscle (ncm) is located internally to the placids of the neck, and is anchored distally to the soft cuticle of the interplacid areas (Additional file 2, Figures 4A-G and 5A-B). When the introvert is everted, the neck circular muscle is expanded, while it is constricted when the introvert is retracted (Figures 7A-B and 8A-F). It should be stressed that this neck circular muscle (ncm) has a fixed position in the adults of *Echinoderes* (compare position in Figure 4A, D, G) as a result of its anchoring points within the interplacid areas of the neck (Figure 7B). However, in contrast, the position of the introvert circular muscle (icm) is not fixed, and is repositioned along the anterior-posterior axis by eversion and retraction movements of the introvert (Figure 8A-F). Additionally, there are four to six fibers extending from longitudinal musculature of the trunk (see below) that attach within the neck region. These muscle fibers attach in between ventrolateral placids 2, 3, 4 and 14, 15, 16 within the interplacid region of the neck (Figures 4A, D, G and 7A-B).

#### Musculature of the trunk

**Figure 9:**
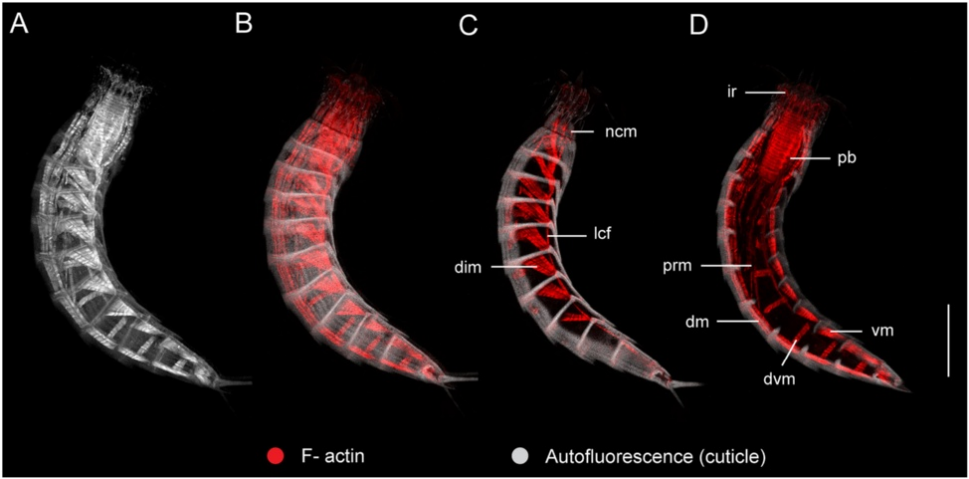
**Musculature within the segmented trunk region of *Echinoderes horni*.** Confocal z-stack projections of phalloidin staining (muscle) and autofluorescence (cuticle) in a single specimen. Lateral views with anterior to the top and ventral to the right side. **(A-B)** maximum z-stack projections of muscle in grayscale **(A)** and red **(B)**. **(C)** z-stack of optical slices on the right side of the medial plane. **(D)** z-stack of optical slices through the medial plane at the level of the pharynx. Ventral, dorsal, dorsoventral and diagonal muscles are segmented. Continuous fibers span multiple segments on dorsal and ventrolateral sides of the trunk, and within the trunk as pharynx retractors. Abbreviations: dim, diagonal muscles; dm, dorsal muscles; dvm, dorsoventral muscles; ir, introvert retractors; lcf, longitudinal continuous fibers; ncm, neck circular muscle; prm, pharynx retractor muscle; vm, ventral muscles. Scale bar, μm.

The musculature of the trunk consists of several different muscle groups, including muscle fibers that span multiple segment margins, and distinct subsets of muscles that are clearly segmental in their orientation and attachment characteristics (Additional file 2, Figures 8A-F and 9A-D). Bilateral sets of individual muscle bands extend longitudinally along the anterior-posterior axis as continuous fibers that span more than one segment, and therefore do not follow a segmental pattern. These muscles include longitudinal retractors of both the introvert (irm) and pharynx (prm), and distinct bundles of fibers that extend from the neck circular muscles to segment 9 along ventrolateral sides of the trunk (Figures 8 and 9). The ventrolateral muscles are distinguished here as lateral continuous fibers (lcf), which appear to attach at their anterior ends to the interplacid areas within the neck (Figure 7A, B) and the first segment (Figures 4A, D, G and 9C), and extend from there to putative attachment sites within consecutive posterior segments of the trunk; their final attachments are made by several fibers within segment 9 (Figures 8A, D, F and 9C). The number of fibers attaching within consecutive trunk segments decreases from anterior-to-posterior. Moreover, in some specimens the lateral continuous fibers (lcf) appear to overlap with diagonal segmental musculature within the first and second segments.

Segmentally arranged musculature is observed within trunk segments 1–10, and includes four distinct groups, in three orientations. Pairs of ventral and dorsal muscles are oriented longitudinally in each segment. There is one pair of ventral muscles (vm), and two pairs of dorsal muscles (dm) that are in subdorsal and laterodorsal positions within each segment (Figures 8C, E and 9A-B, D). In some cases, dorsal muscle fibers may span more than one segment. In segments 1–8, there are pairs of diagonal muscle bands (dim) on left and right lateral sides of the body (Figures 8A-F and 9C). The fibers in each band appear to attach along anterolateral margins of the tergal (dorsal) pachycyclus, extend in an oblique or diagonal pattern, and converge medially to reach the pachycyclus of the following segment near each tergosternal ventrolateral plate junction (Figures 4A, D, G, 8C and 9A-C). The number of diagonal fibers within each segmental muscle band is variable among segments and species, with the first and last muscle bands of the series (segments 1, 2 and 8) typically having fewer fibers than observed within the other segments (Figure 9C). There are distinct pairs of dorsoventral muscles (dvm) in segments 3–10 (Figures 8A-D, F and 9A, B, D). Each dorsoventral muscle band is composed of two fibers. The fibers from each muscle of the pair insert on their respective left and right midventral sides of a segment, and extend dorsolaterally to symmetric attachment sites on the tergal cuticular plate in the center of each segment (Figure 8A, C, D, F). Dorsoventral muscles (dvm) are oriented perpendicular to the anterior-posterior axis, and represent one of the four pairs of segmental muscle bands, along with ventral (vm), dorsal (dm), and diagonal muscles (dim).

**Figure 10:**
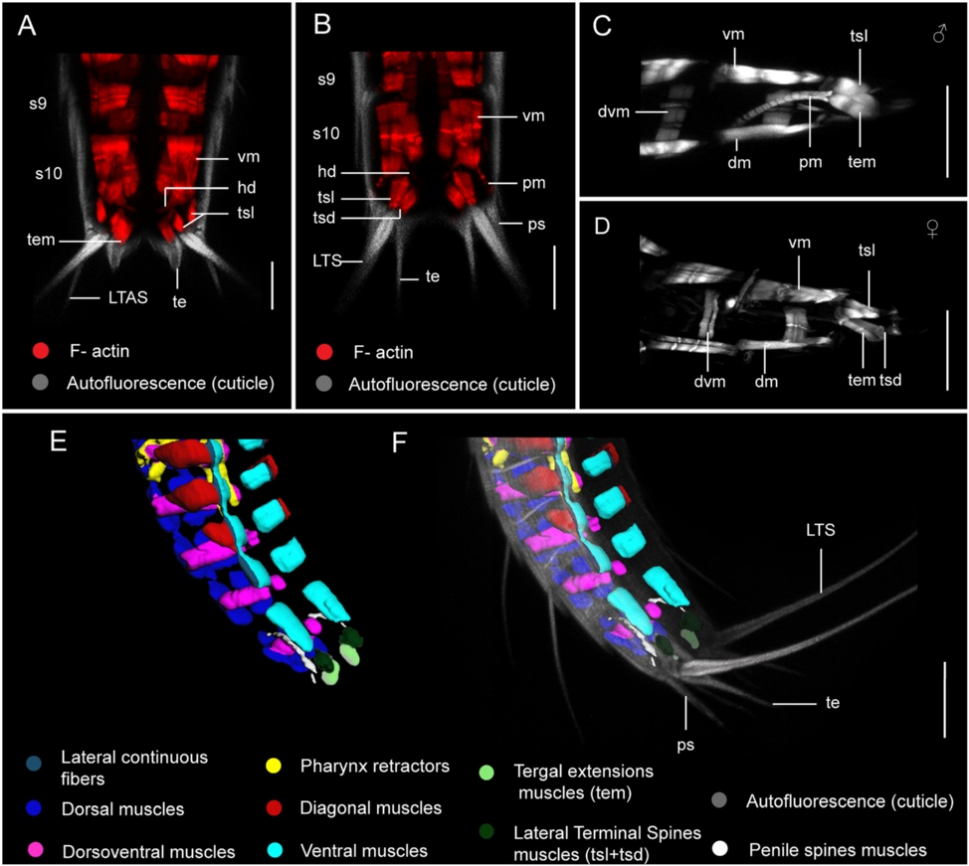
**Musculature of the posterior trunk region and spines in *Echinoderes. *(A-B)** Confocal z-stack projections of phalloidin staining (muscle, red) and autofluorescence (cuticle, grayscale) in segments 9–11 of *Echinoderes horni***(A)** and *Echinoderes spinifurca***(B)** in ventral view, with posterior to the bottom. **(C-D)** Confocal z-stack projections of phalloidin staining in the posterior end of *Echinoderes horni* (**C**, male) and *Echinoderes spinifurca* (**D**, female) in lateral view, with posterior to the right side. **(E-F)** 3D reconstruction of myoanatomy in segments 7–11 of *Echinoderes spinifurca* in lateroventral view with posterior to the right side. Autofluorescence of the cuticle is included in **(F)**. Abbreviations: dm, dorsal muscle; dvm, dorsoventral muscle; hd, hindgut dilator; LTAS, lateral terminal accessory spine; LTS, lateral terminal spine; pm, penile spine muscle; ps, penile spine; s, segment; te, tergal extension; tem, tergal extensions muscle; tsd, terminal spine depressor; tsl, terminal spine levator; vm, ventral muscle. Digits in **(A)** and **(B)** refer to the segment number. Scale bar: 20 μm.

Additional musculature of the trunk is observed in segment 11, and is associated with lateral terminal spines (LTS), and penile spines (ps) of the males (Figure 10A-F). Each lateral terminal spine (LTS) has a pair of terminal spine levator muscles (tsl) that emerge laterally from the base of the spine and extend to the ventral side of the pachycyclus of segment 11 (Figures 8A-F and 10A-F). Another set of muscle fibers form the terminal spine depressor muscles (tsd), which extend from the opposite side of the lateral terminal spine base, and likely act as antagonists of the levator (tsl) muscles (Figure 10B, D). Short tergal extension muscles (tem) are associated with the basal part of each tergal extension, and they also seem to attach to the ventral side of the pachycyclus of segment 11, medial to the attachment of terminal spine musculature (Figures 8A-F and 10A, C, D). The lateral terminal accessory spines (LTAS) on segment 11 in the females of *Echinoderes* do not appear to be supplied with musculature (Figure 10A). In contrast, males have a pair of long, thin, dorsally curved muscles associated with their penile spines (Additional file 2, Figure 10B, C, E-F). A bilateral pair of penile muscles (pm) emerge close to the anterior ends of dorsal longitudinal muscles in segment 10, extend in a posterior direction, and then divide distally into three thin fibers that connect to the basal part of three penile spines on each side of segment 11 (Figure 10C, E, F). Therefore, there are two penile muscles (pm) that operate six penile spines (ps).

#### Gut musculature

The digestive system of *Echinoderes* is subregionalized into a foregut (mouth cone, pharynx, esophagus), midgut and hindgut. Distinct sets of muscles are integrated with each subregion. There is no evidence for segmentation of the musculature associated within or among different subregions of the digestive tract. The myoanatomy of the protrusible mouth cone, which contains the mouth and represents the beginning of the alimentary canal, is described above (section “Musculature of the head”).

The pharynx of *Echinoderes* is a highly complex muscular organ (Figures 3, 4, 5 and 9). The pharyngeal bulb (pb) is a movable cylinder-shaped structure, and is composed of circular muscles (pcm) that alternate with radial muscles (pram) along the length of the bulb (Figures 3C-D, 4C, F, I, 5A-F and 9D). Ten bands of each type are counted, with circular bands being noticeably thinner than the radial bands (Figures 3C-D and 4I). A sphincter (sph) muscle is present at both anterior and posterior ends of the pharyngeal bulb (Figures 3C and 5C). The internal diameter of each sphincter is smaller than the internal diameters of circular and radial muscle bands. The pharynx is always positioned posterior to the buccal cavity; however, its position along the anterior-posterior axis correlates with movements of the mouth cone and introvert, and is therefore observed to vary among specimens according to the position in which they were preserved. Consequently, the pharynx is located inside the trunk when the head (mouth cone plus introvert) is retracted, and it reaches the mouth cone when the head is fully extended (Additional file 1, Figures 4C, F, I and 5A-F).

Around the pharyngeal bulb of *Echinoderes* sp., *E. hispanicus*, *E. horni* and *E. spinifurca* there are eight inner longitudinal muscles (pil). Each one of these muscles (pil) contains two fibers that extend posteriorly from the pharynx to the esophagus and midgut (Figures 2I and 3C). External to the inner longitudinal muscles (pil), there is a conspicuous tulip-shaped muscular sheath (pms) that encloses the pharyngeal bulb (Additional file 1, Figures 2D, 3D and 4I). This sheath is composed of three different muscle groups: prm, psm, and pip. Eighteen pharynx retractor muscles (prm) arranged in pairs on their anterior end, which together form the nine tips of the tulip (Figures 2D, J and 3C). The eighteen, paired retractor muscles (prm) alternate positions with ten pharynx sheath muscles (psm) that do not extend posteriorly beyond the pharynx bulb (Figures 2L and 3C). Two of the ten pharynx sheath muscles occupy the middorsal position (Figure 2L). In addition, nine to ten inner protractor muscles (pip) contribute to the composition of the muscular sheath (pms) enclosing the pharynx. These inner protractor muscles (pip) alternate positions with the sheath muscles and most likely attach to the base of the mouth cone on their anterior ends, and to the base of the pharynx at their posterior ends (Figures 3C and 5D, F).

Four to six outer protractor muscles (pop) appear to attach to the posteriormost end of the pharynx at midventral, midlateral, and laterodorsal positions. These outer protractors (pop) extend from the base of the pharynx toward the head where they likely attach to the body wall area between the introvert and the mouth cone (Figure 3C). These protractors appear J-shaped when the pharynx extends toward the animal’s anterior end, surpassing their insertion point (Figures 3C and 4F, I).

In *E. dujardinii* there are also eight inner longitudinal muscles (pil) surrounding the pharyngeal bulb as described above, although their paired nature cannot be assessed (Figure 5C). Nine inner protractor muscles (pip) with a flame-like shape surround the pharyngeal bulb, and extend well beyond the bulb’s anterior end to attachment sites at the basal end of the mouth cone (Figure 5B-C). In some specimens, one or two of these retractors (pip) appear to be connected with the internal mouth cone ring. The muscular sheath that surrounds the pharynx (pms) of *E. dujardinii* is composed of the inner protractor muscles (pip) that alternate with ten or more pharynx retractors (prm), and ten sheath muscles (psm) (Figure 5B). An undetermined number (2–4) of outer protractors (pop) are present as well, which attach to the base of the mouth cone, and may connect with the external circular muscle (mce) of the mouth cone (Figure 5B asterisk). The general appearance of the sheath in *E. dujardinii* resembles a ‘basket’ around the pharynx. This muscular basket-like structure can be found more or less contracted depending on the position of the pharynx, as observed in a more contracted state when the pharynx is either extended to the mouth cone, or retracted away from the mouth cone.

An additional group of thin longitudinal muscle fibers are observed in all five species. These fibers extend from the middle of the pharynx to segments 3 and 4. They appear to be relaxed when the pharynx is retracted, and not readily visible when the pharynx is extended. These particular muscles have been termed pharynx suspensors (phs) due to their position, morphology and possible function (Figures 3C and 5A, D).

Posterior to the pharyngeal bulb in each species, pharynx retractors (prm) are arranged in four groups, with two oriented in lateroventral positions, and two in laterodorsal positions (Figures 3C and 5E, F). These pharynx retractors (prm) extend into the trunk toward ventromedial and laterodorsal attachment sites within segments 5–8 (Figures 4C, F, 5C, D, F and 8D).

The musculature of the midgut is composed of sixteen inner longitudinal muscles (mil), and sixteen or more thin, outer circular muscles. These filamentous muscle fibers are arranged in an orthogonal grid-like (gg) architecture encircling the midgut intestine (Figure 3D). At the level of the esophagus, the inner longitudinal muscles of the midgut (mil) bifurcate from the eight pairs of inner longitudinal muscles of the pharynx (pil), and extend posteriorly to surround the intestine and the hindgut (esm) (Figure 3D). Between segments 10 and 11, two pairs of short transverse muscle fibers extend from laterodorsal muscles and appear to tether the hindgut laterally. These fibers were designated as hindgut dilator (hd) muscles (Figures 3D, 7F and 9A-B).

## Discussion

### Comparative myoanatomy within *Echinoderes*

Among the five species of *Echinoderes* we examined, there is notable variation in external morphology, although it is primarily limited to differences in the number, length, and presence or absence of small cuticular structures (Table 1). Variations in the external morphology of those species are reflected by relatively minor differences in the internal morphology of their musculature. Most of this variation includes species-specific differences in the head region, such as the shape, length and number of retractor muscles of the introvert and pharynx, as well as the composition of the pharyngeal sheath. Musculature within regions of the neck and trunk is basically similar among species. Overall, comparative myoanatomy within *Echinoderes* is remarkably conserved, and includes the following set of common characters: (Head) two circular muscles and nine pairs of oral style muscles in the mouth cone; ten longitudinal retractors, one circular muscle, and fourteen circular muscle retractors in the introvert; (Neck) one circular muscle; (Trunk) continuous intersegmental fibers among segments 1–9, and segmental pairs of ventral, diagonal, dorsoventral and dorsal muscles within segments 1–10 of the trunk; three pairs of terminal spine muscles and one pair of penile spine muscles in segment 11of male specimens; (Gut) a pharyngeal bulb with ten radial and ten circular muscle bands enclosed in a muscular sheath, including protractor and retractor muscle fibers; an orthogonal grid of midgut muscle fibers, and lateral pairs of hindgut dilator muscles.

Prior to our multi-species investigation with the aid of a confocal laser scanning microscope (CLSM), the myoanatomy of *Echinoderes* was understudied. Microscopical descriptions for the purpose of taxonomic identification or anatomy were conducted with compound light (LM) or transmission electron (TEM) microscopes, and they presented limited coverage of the musculature. From those studies, data are available for a few species, including *Echinoderes aquilonius*, *E. capitatus* and *E. dujardinii*[1,36,44,45]. Upon review, it appears that the anatomy of neck circular muscles, and the number and arrangement of longitudinal, diagonal and dorsoventral muscles in the trunk, is shared among those species. Those results are in agreement with our study. However, there are noticeable differences between the results of previous work and our study regarding the arrangement of muscles in the head, the first trunk segments, and the gut. These particular regions include not only locations in the kinorhynch body plan where multiple types of muscles overlap, but also the most intricate and difficult myoanatomy to interpret, which may introduce both real discrepancies among results and those caused by misinterpretations. Moreover, it is also likely that different *Echinoderes* species from separate studies were examined with different levels of focus and attention to detail. Thus, it is necessary to compare our results with different species from separate studies in order to resolve several issues (Table 2).

**Table 2:** Comparative data on the myoanatomy of Kinorhyncha from this study and available primary literature (references included)

**Species**	***Echinoderes aquilonius***	***Echinoderes capitatus***	***Echinoderes dujardinii***	***Echinoderes *** **sp. *E. dujardinii E. hispanicus E. horni E. spinifurca***	***Antygomonas *** **sp.**	***Zelinkaderes floridensis***	***Pycnophyes dentatus***	***Pycnophyes flaveolatus***	***Pycnophyes kielensis***
**Data source**	TEM	TEM	LM	CLSM	CLSM	TEM	TEM	TEM	TEM, CLSM
**Mouth cone**	-	3 circular muscles	-	18 longitudinal, 2 circular muscles	16 longitudinal, 8 short basal and 2 circular muscles	18 longitudinal muscles	2 circular cell basal to oos, 7 circular muscle cells basal to ios	-	1 circular muscle cell basal to oos, 7 circular muscle cells basal to ios
**Introvert**	16 outer retractors 12 inner retractors	10 retractors reaching S.6	-	10 retractors reaching S.6, Introvert circular muscle	16 outer retractors reaching S.8	-	10 head retractors	-	10 head retractors
**Neck**	-	-	1- 5 circular muscles	1 circular muscle	2 circular muscles: inner outer	-	-	-	-
**Trunk**	-	-	Longitudinal: dorsal, ventral, and additional continuous fibers, Diagonal: 8 pairs Dorsoventral: S.3-11	Longitudinal: Ventromedial + Dorsolateral and additional continuous fibers, Diagonal: 8 pairs Dorsoventral: S.3-11	Longitudinal: Ventrolateral + Dorsolateral Diagonal: 9 pairs Dorsoventral: S.1-11	-	-	-	-
**Spines**	-	-	-	3 pairs LTS + 1 pair TE muscles 1pair penile spines muscles	Midterminal spine muscles triangle- shaped	-	-	-	-
**Pharynx**	Circular lumen	-	-	Circular lumen, 10 radial + 10 circular fibers	Circular lumen, 14–15 circular fibers alternate with inhomogeneous structures, pre- and postpharyngeal sphincters 10 protractors, Pharynx sheath present	Circular lumen, 15 radial +15 circular fibers,2 prepharyngeal 1postpharyngeal sphincters	Triradiate lumen	Triradiate lumen	Triradiate lumen
							13 radial + 14 circular muscle cell	Circular and radial muscular fibers	13 radial + 14 circular muscle cell
							1postpharyngeal sphincter	1postpharyngeal sphincter	1postpharyngeal sphincter
**Midgut**	12-16 longitudinal muscles	-	-	Muscular grid: Inner longitudinal (16) + outer circular (+16)	Muscular Grid: Inner longitudinal + outer circular	Muscular Grid: Inner longitudinal + outer circular	Muscular Grid: inner longitudinal + outer circular	-	Muscular Grid: inner longitudinal + outer circular
**Hindgut**	-	-	-	1-2 pairs short dilator muscles	1 dilator muscle	6 dilators: 2 caudal + 2 dorsal 1 frontal 1 circular	4 dilators: 2 caudal + 2dorsal; 2dorsal + 1 ventral transversal muscle cell	-	4 dilators: 2 caudal + 2dorsal; 2dorsal + 1 ventral transversal muscle cell
**Reference**	[1]	[33]	[41,42]	This study	[26]	[30]	[30]	[38]	[5,27,30]

Myoanatomical features observed using transmission electron microscopy (TEM), light microscopy (LM) or confocal laser scanning microscopy (CLSM). Abbreviations: LTS, lateral terminal spines; S, segment; (-) data not available.

Kristensen and Higgins [1] described retractor muscles in the introvert of *E. aquilonius* that consisted of two sets, one composed of sixteen outer retractors, and the other set with twelve inner retractors. Their descriptions differ from what we have observed in the studied species of *Echinoderes*. In *E. aquilonius*, the sixteen outer retractors appear to extend from segment 9, pass through the forebrain, and attach to the anteriormost end of the introvert [1]. We identified ten introvert retractors in each of five species from approximately one hundred specimens. These ten retractors also extend through the forebrain, however, they attach at the level of the first row of spinoscalids and alternate positions with them. Kristensen and Higgins [1] may have misidentified some protractors of the pharynx as introvert retractors, leading to the wrong assignment and a higher total count. Despite these differences in number and arrangement, our introvert retractors may be the “outer retractors” described by Kristensen and Higgins [1]*.* We also identified 18–20 pharynx retractors, which may correspond to the “inner retractors” of *E. aquilonius*, although our count differs again from the twelve described [1]. These and other differences likely stem from our application of fluorescent markers and CLSM, which enabled staining and visualization of complete sets of muscles in multiple focal planes. Additionally, we labeled cellular structures of the brain, introvert and pharynx for contextual landmarks, and the ability to discriminate between distinct muscle fibers within highly complex regions of kinorhynch myoanatomy. As a result, we reevaluated previous ambiguities, such as *inner* and *outer* retractors [1], in order to recount and reassign organ-specific fibers and muscle groups (e.g. introvert distal retractors, pharynx outer protractors).

The number and arrangement of mouth cone muscles reported herein appears different from what was found in *E. capitatus*[36], where three circular muscles were described, without reference to any longitudinal fibers. In contrast, we clearly identified two circular muscles, and nine pairs of short longitudinal muscles in each of the five echinoderid species. Nebelsick [36] may have easily overlooked these longitudinal muscles due to their small size and position within the mouth cone. Regarding the number of mouth cone circular muscles, Nebelsick [36] shows three individual muscles in *E. capitatus* (see Figure 1 therein), however we suggest that based on their positions they are actually two circular muscles which correlate with what we found in our study.

Circular muscles of the neck are commonly identified in studies of *Echinoderes*. Zelinka [44] described a single muscle in the neck region, whereas Remane [45] described five rings in *E. dujardinii.* Our results corroborate the observations of Zelinka [44], and suggest that Remane [45] could have misidentified circular muscles from the introvert and mouth cone as additional neck musculature. Kristensen and Higgins ([1], see Figure twenty-seven therein) presented a detail of circular muscle attachment in the neck of *E. aquilonius*, although it was not described. We identified a similar arrangement between the neck circular muscle and its proximity to the interplacid areas (Figure 7B). This particular attachment site, where the neck circular muscle is oriented along the anterior end of the placids, and attached to softer interplacid cuticle, was confirmed by additional evidence (R. M. Kristensen, personal communication).

The presence of continuous longitudinal fibers in the trunk of *Echinoderes* is also supported by TEM images of *Echinoderes cantabricus*, but was not discussed by Gª Ordóñez et al. [37]. Accordingly, Remane [45] described a similar pattern in *E. dujardinii* as the splitting of longitudinal muscles in the anterior end of the trunk. This ‘splitting’ of muscles described by Remane [45] most likely corresponds to the longitudinal continuous fibers that fan out within the trunk of *Echinoderes* species investigated here. The sixteen longitudinal midgut muscles of the orthogonal gut grid observed in *Echinoderes* species in our study also support previous observations by Kristensen and Higgins [1] who described 12–16 longitudinal muscles in the trunk of *E. aquilonius*.

### Comparative myoanatomy of Kinorhyncha

Within Kinorhyncha, there have been TEM studies of internal morphology and ultrastructure, with details of myoanatomy in several genera: *Zelinkaderes floridensis*[33], *Pycnophyes dentatus*[33], *Pycnophyes flaveolatus*[41], *Pycnophyes greenlandicus*[1], and *Pycnophyes kielensis*[5,33]. Previous descriptions of musculature by confocal microscopy have also been presented, although they were limited to one cyclorhagid species, *Antygomonas* sp. [29], and one homalorhagid species, *P. kielensis*[5,30]. Thus far, most of the attention has been on longitudinal and segmental musculature of the trunk, with only one description of myoanatomy in the head and neck [29]. Unfortunately, all of the specimens in that study had their introvert fully or partially retracted, precluding detailed resolution of anterior musculature, such as important differences between the anterior radial closing system of Echinoderidae and the bilateral closing system of species within Antygomonidae. Yet, when considering all of the available information, *Antygomonas* sp. [29] is the most appropriate model for direct comparison with *Echinoderes*.

We found eighteen outer oral style muscles in each species of *Echinoderes* studied here, representing one pair of muscles for each outer oral style. Müller and Schmidt-Rhaesa [29] reported sixteen outer oral style muscles, which would indicate that one of the nine oral styles of *Antygomonas* sp. does not have a pair of muscles. Additionally, we did not find any of the eight basal mouth cone muscles described in *Antygomonas* sp. ([29]; see Figure four A therein). These short, single mouth cone muscles may be exclusive to Antygomonidae, or perhaps they are attachment sites for the sixteen mouth cone muscles. We did identify 14–16 short introvert circular muscle retractors (icmr) in each species of *Echinoderes*, which are most likely comparable to these singular fibers that “stretch toward the first circular ring” in the neck region of *Antygomonas* sp. [29]. Although they were not characterized as circular muscle retractors, we consider them as such because they appear to extend from the introvert circular muscle toward the body wall in the first segment, which is comparable to the condition of ‘icmr’ fibers in species of *Echinoderes*. We have clearly identified fourteen introvert circular muscle retractors in *Antygomonas paulae* (unpublished observations, Herranz and Boyle). The outer retractors (or) of *Antygomonas* sp. [29] should also be considered comparable to the introvert distal retractors in *Echinoderes*. Müller and Schmidt-Rhaesa [29] observed two mouth cone circular muscles, as well as the introvert and neck circular muscles within *Antygomonas* sp., which match our observations in *Echinoderes*.

Within the trunk region, intersegmental and segmental muscle bands most likely occur in all kinorhynchs. However, diagonal muscles have been described almost exclusively for cyclorhagids [44,45], with only one report of diagonal bands in a homalorhagid species, *Pycnophyes calmani*[50]. The diagonal muscles of *Echinoderes* show a clear segmental pattern along the trunk, while in *Antygomonas* sp., each of the first three diagonal muscles span two segments [29]. These differences could arise from variation in musculature of the neck and first trunk segments associated with radial vs. bilateral closing systems. The dorsoventral muscles are the only segmental muscles of the trunk that do not attach to pachycycli, and therefore they do leave conspicuous cuticular scars [1,29,44,45]. These muscles are absent in the first and second ring-like segments of *Echinoderes*. Other genera within Echinoderidae, and Kinorhyncha, vary as to whether segment 2 is composed of one or two sternal plates, which may correlate with changes in the arrangement and attachment of musculature in the first two segments. As previously mentioned, further detailed comparative studies of kinorhynch closing systems are needed to address such questions.

Myoanatomy of the pharyngeal bulb has been described in several genera and species [1,29,33]. Within the pharynx, there are notable differences in the shape of the lumen, and the number of circular and radial muscle fibers. In Cyclorhagida, including *Echinoderes*, the shape of the pharyngeal lumen is primarily circular, while in Homalorhagida the lumen exhibits a triradiate, or inverted Y-shaped, configuration [20,21]. Since triradiate pharynges are most likely homologous in Ecdysozoa, this would suggest that Homalorhagids exhibit the primitive kinorhynch gut architecture [20]. Yet we remain cautious, as the first molecular phylogeny of kinorhynchs did not recover monophyly for either Cyclorhagida or Homalorhagida [51], and other evidence suggests that a triradiate pharynx “cannot be ancestral in the cycloneuralians” and likely evolved in parallel multiple times [21]. Regarding variation in the number of alternating circular and radial fibers among genera, there are fifteen in *Zelinkaderes*, thirteen-to-fourteen in *Pycnophyes* and fourteen-to-fifteen circular fibers alternating with inhomogeneous structures in *Antygomonas*[29,33,35] (Table 2). Our results show ten circular and ten radial fibers in *Echinoderes*. This arrangement appears highly conserved among species, and may become an important diagnostic character for the genus. It is not known whether this arrangement of pharyngeal muscle fibers is shared within Echinoderidae.

Musculature associated with terminal spines has been reported in *Pycnophyes kielensis*[30], and a strong, paired muscle was described at the base of the midterminal spine in *Antygomonas* sp. [29]. Although Müller and Schmidt-Rhaesa [29] did not state it, their data also suggest there could be muscles in the bases of lateral terminal spines in *Antygomonas* sp. Additional kinorhynch genera with midterminal and lateral terminal spines include *Centroderes, Zelinkaderes* and *Semnoderes*, however spine musculature has not yet been investigated within or among these genera. The five species of *Echinoderes* in our study share a similar arrangement: muscles are distinctly associated with lateral terminal spines, but not with lateral terminal accessory spines, which are rigid and immobile in live animals. The presence of penile spines in male kinorhynchs is shared by *Cephalorhyncha*, *Dracoderes, Echinoderes*, *Fissuroderes*, *Meristoderes and Pycnophyes*. Of those genera Myoanatomical data are only available for species of *Echinoderes* and *Pycnophyes*, and until now, penile-spine musculature was not identified in either genus. Our results show conspicuous muscles associated with penile spines in all five *Echinoderes* species, and we predict that similar muscles will be identified in species from each of the genera where penile spines are present.

### Interpretations of functional myoanatomy in *Echinoderes*

With the exception of smooth muscles of the intestine, the sarcomeres of kinorhynch myofibrils have a predominantly cross-striated arrangement [1,29,30]. This enables rapid muscle contraction associated with feeding and locomotion [20]. Both feeding and some forward locomotion behaviors are produced by eversion and retraction of the introvert, which is characterized by complex myoanatomy within head and neck regions (Additional file 3; [1]). Not only are well-developed circular and retractor muscles of the head and neck involved in movements of the introvert, but they also exemplify the unsegmented radial symmetry of the mouth cone, introvert and cuticular ring of placids, which support previous suggestions that there are no true segments in the head or neck region of kinorhynchs [1,14,52,53].

Here, we propose a model for retraction of the introvert by combined action of three sets of muscles in *Echinoderes*: introvert retractors, introvert circular muscles, and introvert circular muscle retractors (Figure 6). When the introvert is everted, introvert retractors (ir or idr + ipr, respectively), circular muscles (icm), and circular muscle retractors (icmr) are relaxed and stretched (Figure 6A). Ten introvert retractor muscles are attached at the level of the primary spinoscalids and alternate with them in a radial arrangement. During contraction, introvert retractors cause the primary spinoscalids to fold inward, followed by the remaining scalids (Figure 6B). Synchronous contraction of introvert retractors and the relaxation of dorsoventral muscles within the trunk [1], combine to initiate retraction of the introvert. Musculature is absent within primary and other scalids, which implies that scalids cannot move independently from each other, and thus move passively during relocation of the head. The introvert circular muscle (icm) then contracts, reducing the volume and diameter of the introvert, enabling it to pass through the neck. At the same time, the introvert circular muscle retractors (icmr) contract, which supplements the introvert retractors, leading to retraction of the introvert (Figure 6C).

Dorsoventral muscles of the trunk play an important role during extension of the introvert. The contraction of dorsoventral muscle fibers causes an increase of internal pressure within the reduced body cavity, acting as a hydrostatic skeleton to extend the introvert [20,25]. Once the introvert is everted, the mouth cone protrudes. Internal hydrostatic pressure within the body cavity may not be enough to fully extend the mouth cone. However, the mouth cone has an intimate positional relationship with the pharynx. When the pharyngeal protractor muscles contract, the pharynx moves in an anterior direction pushing the mouth cone outward (see below). When the pharynx is retracted toward the trunk by contraction of pharyngeal retractor muscles, the mouth cone is also retracted.

Typical food items found within the gut of kinorhynchs include bacteria, diatoms, algae and detritus [35,36,44,54]. The articulated outer oral styles may be used as ‘forceps’ for grasping, manipulating, and ingesting those and other food items [55]. In order to feed this way, muscles at the base of the outer oral styles (osm) are most likely antagonistic with an external circular muscle (mce) of the mouth cone (Figure 3A-B). When the paired, longitudinal oral style muscles contract, the external circular muscle relaxes and the oral styles are straightened and extended (Figure 3A). Conversely, when the external circular muscle contracts, the paired oral style muscles stretch and the outer oral styles bend (Figure 3B). The radial distribution of oral style muscles, as well as introvert retractors, indicate a correlated function. As previously suggested, this combination of longitudinal and circular muscles may enable limited motion of the outer oral styles [33]. Their main function could be to sense and respond to external stimuli, which is in agreement with TEM studies that show a sensory cell at the base of each outer style [1,32,36]. However, both sensory and grasping functions may be combined. It would seem reasonable that the animal must detect and discriminate what is ingested. The inner oral styles are not articulated, and show only a circular muscle around their base. This may function to selectively open the mouth in the presence of food particles [1,33,55]. Furthermore, ciliated receptor cells and terminal pores have been confirmed in each of the inner oral styles, suggesting there may also be a sensorial feeding-related function for these structures [1,38].

The neck acts as an anterior closing apparatus of the body by synchronous movements of alternate hard (placid) and soft (interplacid) cuticular elements [44,45]. The radial symmetry of this ‘closing system’ is shared by all genera within Echinoderidae. In *Echinoderes,* the neck circular muscle attaches to soft interplacid cuticle lining the distal perimeter of the neck region (R.M Kristensen personal communication). When the introvert is retracted, the placids rotate toward the center of the neck on hinge-like articulations between the placids and the first trunk segment (Figure 7A and 1H). When the introvert is everted, the neck circular muscle is relaxed to its widest diameter along interplacid attachment sites. In this relaxed position, the hard placids are ‘flipped open’ and aligned with the anterior-posterior axis (Figure 7B). Rotation of the placids is coordinated with contraction of longitudinal fibers (lcf) that are attached to interplacid sites of the neck (Figure 7B). When the introvert is completely withdrawn, the circular muscle is contracted to its smallest diameter, soft interplacid cuticle is pulled toward the center of the neck, and the placids become tightly juxtaposed to close off the anterior of the trunk (Figures 1H and 7A). Consequently, the neck and introvert are functionally linked and interdependent [1,53]; however, while circular muscles of the mouth cone and introvert become repositioned along the anterior-posterior axis, the neck circular muscle maintains a fixed position. Both the introvert and neck circular muscles are ultimately involved in introvert retraction and closure of the anterior trunk in *Echinoderes*, they are most likely independent systems, where introvert retraction is dynamic and often partial without closing off the trunk body. Contrary to this, Müller and Schmidt-Rhaesa [29] suggested that both muscles primarily control the closing system (neck) of *Antygomonas* sp. However, each of their respective closing systems exhibits a distinct morphology and thus requires a more thorough investigation.

The trunk has paired sets of longitudinal, dorsoventral and diagonal muscles that enable these animals to perform a range of movements. In *Echinoderes*, the relaxed body plan exhibits a curvature of the dorsoventral axis along the trunk (Figure 1A-E). Segmented dorsal and ventral longitudinal muscles may act as antagonists to produce this curvature. And paired sets of continuous fibers that span several segments likely contribute to the high degree of trunk flexibility observed in living specimens (Additional file 3). Pairs of dorsoventral muscle bands join tergal (dorsal) and sternal (ventral) plates in segments 3–10. These muscles are thought to be derived from circular muscles [1,56]. However circular muscles are reduced or absent in the trunk of kinorhynchs, and a reasonable hypothesis for the origin of dorsoventral musculature is lacking. Yet, as with other muscle types, dorsoventral muscle bands are integral to kinorhynch locomotion and feeding. The contractions of dorsoventral fibers pull each plate toward the center of the body, which increases the internal pressure of the trunk and contributes to eversion of the introvert. To our knowledge, contraction of dorsoventral musculature has not been characterized in *Echinoderes*, or Kinorhyncha. We suspect that during introvert eversion, contraction of these muscles would proceed sequentially from posterior to anterior, analogous to peristaltic movements in soft-bodied invertebrates. Synchronous contraction may not be as effective, impeding anterior movement of the pharynx and mouth cone. Diagonal muscles are only present from segments 1–8, and contribute to lateral movements of the animal within that trunk region [44,45]. Because they are not present in segments 9–11, the posterior trunk is less flexible, which may confer an unrecognized functional stability associated with sexual reproduction.

We did not detect musculature within dorsal and lateroventral spines. Müller and Schmidt-Rhaesa [29] described small, triangular paradorsal muscles at the base of middorsal spines in *Antygomonas* sp. that could be responsible for the movements of those spines. However, subsequent studies revealed that actin filaments in circumciliary microvilli of paradorsal sensory spots were misinterpreted as muscles [20,35]. Among species of *Echinoderes*, we observe a similar pattern of ‘triangular’ labeling in trunk positions corresponding to sensory spots, which appears to support the alternative explanation for the presence of actin filaments in microvilli. Musculature associated with lateral terminal spines, tergal extensions and penile spines appear to be specializations of the ventral and dorsal longitudinal musculature [29]. It is not known whether segmented ventral or dorsal muscles act simultaneously or independently with spine movements. Lateral terminal spines are directly controlled by antagonistic pairs of short muscles. Lateral terminal accessory spines do not have musculature and thus any observed movement of those spines is passive. Interestingly, penile spines are connected to long, thin muscle fibers that trifurcate distally, with individual fibers attaching to each of three spines on left and right sides of segment 11. Due to size and location, it is not yet clear whether each penile spine is capable of independent movements. Although the function of a penile spine has been questioned [1,14,35] detection of penile-spine-specific muscle fibers indicates a potential for distinct movements of each spine during the process of mating.

The pharynx is composed of a pharyngeal bulb, encircled by multiple fibers with different but integrated functions. It is the most complex muscle system in *Echinoderes*. Radial and circular muscle fibers of the pharynx bulb are reported in many studies ([1,33,44] and references therein). Contraction of radial fibers increases the bulb’s lumen diameter, while contraction of circular fibers decreases the lumen diameter. Together, antagonistic muscle coordination enables the pharynx to function as muscular sucking pump [1,20,25,57]. The bulb’s anterior sphincter is likely to have a selective function as food moves into the buccal cavity. The posterior sphincter may regulate passage of mechanically digested food into the midgut, and prevent backflow from the midgut during changes in body pressure [35]. Pharynx retractors move the bulb in a posterior direction when the head is retracted into the body. Outer pharyngeal protractors (pop) ‘pull’ the pharynx bulb out of the trunk when the introvert is everted. The inner protractors (pip) also move the pharynx in an anterior direction, further toward the mouth cone. The process of protraction may be very fast, due to a combination of pharyngeal musculature (pop + pip) and the increase of internal body pressure from dorsoventral muscles (dvm) of the trunk. The pharynx suspensor muscles (phs) could act to maintain the position of the pharynx bulb, and prevent twisting, when the pharynx is retracted within the trunk. Additional musculature surrounding the pharynx bulb may assist in the movement of gut contents, such as antagonistic contraction of longitudinal and radial fibers as suggested by Neuhaus [33]. The orthogonal grid of longitudinal and circular muscles surrounding the midgut implies that digestion may be enhanced, in part, by peristalsis. This would enable particles to be displaced toward the hindgut where transverse dilatator muscles regulate defecation [35]. A circular muscle acting as an antagonist to these posterior dilatators was described in *Centroderes* sp. and *Zelinkaderes floridensis*[33]. We did not identify a hindgut circular muscle in *Echinoderes.*

### Comparative myoanatomy between kinorhynchs and closely related groups

Based on morphological characters, Kinorhyncha, Priapulida and Loricifera are grouped together as the Scalidophora [11,12,33]. Molecular analyses have suggested an alternative hypothesis, where kinorhynchs and priapulids form a monophyletic group, excluding Loricifera [13,58]. However, very few molecular characters have been sampled for Loricifera, and with improved molecular and taxonomic sampling, loriciferans may become repositioned within Scalidophora. Thus far the most consistent interpretation is that Scalidophora (kinorhynchs + priapulids ± loriciferans) is recognized as the most basal branch within Ecdysozoa [7,15,16,19,59]. Accordingly, comprehensive descriptions of myoanatomy in *Echinoderes* and other kinorhynch genera will broaden our understanding of how different types and patterns of musculature evolved within Scalidophora, and by extension, Ecdysozoa. Yet our results also imply that new mysteries have surfaced regarding putative ancestral patterns of musculature in the lineage of animals preceding the scalidophorans. For instance, there are undeniable contrasts in the size, symmetry and complexity of scalidophoran body plans that are reflected in their respective myoanatomy. Most notable is the obvious pattern of segmentation in Kinorhyncha that is apparently absent in Priapulida and Loricifera.

Comparatively, kinorhynchs, priapulids and loriciferans have an eversible head that facilitates locomotion, feeding, and protective behavior. In kinorhynchs, the radially symmetric mouth cone and introvert are equipped with unique arrays of oral styles and rings of scalids that likely overlay an original bilateral symmetry, as observed within the trunk of all kinorhynch species [38]. The development of such radial symmetry at the anterior end is most likely an adaptation to their burrowing mode of life, which involves uniform contact with sediments [38]. Although members each of these phyla ‘burrow’ in sediments, the segmented trunk of kinorhynchs also differs from the body plans of priapulids and loriciferans, which are essentially non-segmented vermiform or ovular, respectively. Thus, different numbers and arrangements of trunk muscles are not directly comparable among these three animal groups. Nevertheless, Kristensen and Higgins [1] suggested that, although distinctly segmented, longitudinal muscles in the trunk of kinorhynchs might be homologous to a layer of longitudinal muscles in the body of priapulids. This inference would appear to be supported by a study from Rothe and Schmidt-Rhaesa and Schmidt-Rhaesa and Rothe [5,30], who observed continuous longitudinal muscles in the trunk of a juvenile kinorhynch prior to their segmental pattern in the adult, implying a developmental transition or the presence of a particular non-segmented muscle type in ancestral, adult kinorhynch taxa. Such homology would have to be demonstrated by developmental studies that identify a similar cellular origin and similar genetic specification mechanism for distinct muscle types in each group of animals, which is thus far not available. In our opinion, direct comparisons of myoanatomy among Kinorhyncha, Loricifera and Priapulida are only possible within their head and neck regions, at least as far as our methods have revealed.

In loriciferans, the distal part of the mouth cone is arranged in a hexaradial pattern, while it is pentaradial in priapulids and kinorhynchs [42]. However, the internal arrangement of musculature does not reflect their respective patterns of mouth cone symmetry. In Kinorhyncha, there are 8–9 pairs of short longitudinal muscles in the mouth cone [29,33], whereas there are eight individual muscles in Loricifera [39]. Based on their arrangement, Neves et al. [43] consider these muscles to be homologous in both phyla. Comparable muscle groups have not been found in priapulids. The radial arrangement of introvert scalids is roughly pentagonal in priapulids and kinorhynchs. Priapulids show twenty-five longitudinal rows of scalids in the introvert [40], whereas the kinorhynch introvert typically bears individual rings of 10–20 scalids each. Loriciferans exhibit a highly variable arrangement of introvert scalids among genera and species, as well as among larval and adult stages. And, introvert scalid musculature is intrinsic in loriciferans, yet completely extrinsic in kinorhynchs and priapulids, resulting in the passive movements of rows or rings of scalids in the latter two taxa [1,42]. Furthermore, there are several longitudinal and circular muscles within the introverts of both priapulids and loriciferans; however, the arrangement is different in each phylum, with a densely packed grid-like pattern of body wall muscles in priapulids, and a net-like pattern composed of few circular muscles and notably thin longitudinal fibers in loriciferans that appear to be associated with the anteriormost rows of scalids [43]. Kinorhynchs lack a grid or net-like arrangement of muscles in their introvert, and instead show a single introvert circular muscle that is integrated with several retractors.

Introvert retractors are generally referred to in the literature as inner and outer retractors, attaching to the introvert wall on both sides of the brain in each of these three phyla [1,35,39,40,42]. This arrangement has been suggested as one of several apomorphic characters for grouping scalidophorans together [21]. However, further comparative analyses of introvert musculature indicate there are several important taxon-specific differences. The so-called inner retractors of kinorhynchs are in fact part of the pharyngeal bulb musculature, and participate in retraction of the pharynx and the mouth cone, not the introvert. And the outer retractors of *Echinoderes* are introvert retractors, and therefore inner and outer retractors are assigned to separate organs. From a recent cytochemical study of myoanatomy in the loriciferan *Nanaloricus* sp., there is some evidence that dorsal and ventral longitudinal retractors of the head might correspond to the outer retractors, while the posterior part of the mouth cone retractors could correspond to the inner retractors described for *Nanaloricus mysticus* by TEM [42,43]. However, there is an intricate system of mouth cone and buccal tube retractors that occupy a similar position, and therefore a definitive homologization is not yet possible without co-labeling musculature and nucleic acids or neurites of the loriciferan brain, or further comparative studies at the ultrastructural level. Regarding the unusual passage of muscle fibers through tissues of the brain, we did find a similar pattern in *Echinoderes* to what has been found in loriciferans, supporting previous observations [1,39,42]. In priapulids, inner and outer retractors were designated as short and long, and were not given positional correlations relative to brain structure [40]. So it appears that more information is required to confirm spatial relationships between organ-specific retractors and cycloneuralian brain architecture within loriciferans and priapulids. Fibers that extend along inner and outer sides of a collar-shaped brain in different taxa, but connect with non-homologous organs, may simply represent convergent solutions to the same problem. If there is a correspondence between inner (pharynx/mouth cone) and outer (introvert) retractors confirmed by co-labeling experiments in all three phyla, it may prove to be an apomorphy of Scalidophora. Until then, designating the arrangement of ‘inner and outer retractors’ as a unifying character should be treated with caution.

Loriciferans and kinorhynchs also share a distinct neck region with a single, thick circular muscle [1,43,44]. In priapulids, only larvae exhibit a well-developed neck region, whereas in adults the introvert and trunk typically join each other directly [40]. And although there is some external similarity between a priapulid larva and adult loriciferans, there is no circular muscle associated with the neck region of larval priapulids. Furthermore, larval priapulids can retract their head and neck internally within the trunk for protection, in contrast to the non-retractable neck regions of kinorhynchs and loriciferans. This could indicate that the neck regions are non-homologous in Priapulida and in Kinorhyncha.

In Loricifera, the pharynx is composed of myoepithelial cells with radial fibers [20,39,42]. Within Cycloneuralia, a myoepithelial pharynx is only shared by loriciferans and nematodes, with putative secondary losses in priapulids and kinorhynchs, however this type of pharynx most likely evolved independently several times [20,21,33]. The pharynx in priapulids and kinorhynchs includes both an epithelium and a muscle layer [1,20,21].

Nematomorphs typically are considered cycloneuralians and hence closely related with the scalidophorans. The adult has an extremely long and slender vermiform body plan, with a reduced digestive system and a rounded anterior end often lacking a distinct head [21,25]. Adult nematomorph features are not readily comparable with scalidophorans, however, an endoparasitic larval form is equipped with an introvert and retractable proboscis [21,60]. Muscle groups that operate the introvert and proboscis (mouth cone) are somewhat comparable to muscle groups in the head regions of priapulids, kinorhynchs and loriciferans, although based on a single study by CLSM for the nematomorph larva of *Gordius aquaticus*[60]. Musculature within the larva’s anterior trunk include six introvert retractors, and six proboscis retractors, with none of those retractors associated with circular muscles, which appear to be entirely absent. Some similarity between loriciferans and nematomorphs is observed regarding the position and function of proboscis retractors in the *Gordius* larva and the buccal tube retractors of adult *Nanaloricus* sp. [43]. Moreover, the oblique muscles described in the *Gordius* larvae can have the same function as the retractors of the introvert in kinorhynchs. Overall, the numbers and arrangements of particular muscle groups in this nematomorph larva are not shared with scalidophorans, especially when comparing introvert circular muscles that are common to priapulids, kinorhynchs and loriciferans but absent in Nematomorpha. It appears that the muscular organ systems of Scalidophora are relatively distinct from other cycloneuralian taxa, contributing to their putative monophyly.

## Conclusions

The myoanatomy of *Echinoderes* is highly conserved. The following muscle groups were identified within each of five species: (i) two mouth cone circular muscles, and nine pairs of oral styles muscles in the mouth cone; (ii) ten introvert retractors, one introvert circular muscle, and fourteen introvert circular muscle retractors in the introvert; (iii) one neck circular muscle; (iv) ventral and dorsal longitudinal muscles within segments 1–10, longitudinal continuous fibers spanning subsets of segments 1–9, diagonal muscles in segments 1–8, and dorsoventral muscles in segments 3–10, all of them in the trunk; (v) a pharynx bulb composed of ten radial and ten circular muscle fibers, a sheath of pharyngeal protractors and retractors surrounding the pharynx, an orthogonal grid of longitudinal and circular fibers surrounding the intestine, and a minimum of one pair of hindgut dilators; (vi) three pairs of terminal spine muscles, and one pair of penile spine muscles in segment 11. Between species of *Echinoderes*, minor differences were observed among introvert retractor muscles and the composition of pharyngeal sheath musculature.

Within Kinorhyncha, there are common myoanatomical traits: introvert scalids are not supplied with muscles; the pharynx bulb is surrounded by a complex array of retractors and protractors forming a conspicuous muscular sheath. There are both segmented and unsegmented muscles within the trunk. Dorsal or lateroventral spines are not associated with musculature. All terminal spines are associated with musculature except for lateral terminal accessory spines (LTAS). Kinorhyncha, Loricifera and Priapulida have common sets of anterior retractor muscles, and should not be utilized as phylogenetic characters since they do not correspond in number, arrangement, and attachment patterns. They are most likely convergent adaptations to a shared burrowing life style. Within Scalidophora, the muscular organ system is comparatively more similar between kinorhynchs and loriciferans.

This study provides the first comprehensive investigation of myoanatomy in Kinorhyncha by CLSM and three-dimensional reconstruction, with comparative descriptions of the form and function of muscles systems in the head, neck and trunk regions of *Echinoderes* (Echinoderidae, Cyclorhagida). Our results build upon previous investigations by transmission electron microscopy, and have begun to address questions about the origins of complex myoanatomy in kinorhynchs, and closely related taxa. In the future, important insights must come from genomic and/or transcriptomic sequence analysis, characterization of gene expression patterns within muscle tissues and other organ systems, and a thorough study of early development in Kinorhyncha, the embryos of which remain elusive.

## Materials and methods

### Animal collection and fixation

Adult specimens of five echinoderid species were utilized for this project: *Echinoderes horni* and *Echinoderes spinifurca* were collected from the Western Atlantic Ocean along the southeast coast of Florida, USA; *Echinoderes dujardinii*, *Echinoderes hispanicus* and *Echinoderes* sp. were collected from the Eastern Atlantic along the southern coast of Portugal (Table 3). Benthic marine sediments were sampled from the intertidal zone with a shovel, or obtained offshore by deployment and retrieval of a Higgins meiobenthic dredge or an anchor dredge on board two different research vessels (RV Sunburst, Smithsonian Marine Station at Fort Pierce, Florida; RV Pagrus, CCMAR, Faro). Sampling depths ranged from shallow intertidal to 45 meters, and sediment types varied in composition among sampling stations (Table 3). Kinorhynchs were extracted from sediments following the “bubbling and blot” technique of Higgins [31,52]. Live specimens were isolated, identified, and fixed with 4% paraformaldehyde (Electron Microscopy Sciences) in filtered seawater or 0.1 M phosphate buffered saline (PBS), overnight at 4°C. Following fixation, specimens were washed with multiple exchanges of 0.1 M PBS, and stored at 4°C in a solution of PBS containing 0.05% sodium azide (NaN_3_) to prevent microbial contamination.

**Table 3:** **Summary of sampling and collection data for five species of *Echinoderes***

** Species**	**Locality**	**Date**	**Geographical coordinates**	**Depth**	**Sediment type**	**Collecting gear**
*E. dujardinii*	Ramalhete Ria Formosa (Portugal)	May 3rd, 2012	37° 00.00 N 7° 58.63 W	Intertidal	Mud with *Zostera* sp.	Shovel
*E. hispanicus*	Off shore Albufeira (Portugal)	April 28th 2012	36° 57.83 N 8° 12.63 W	45 m	Shell gravel	Higgins meiobenthic dredge/Van Veen grab
*E. horni*	4 miles station Fort Pierce (USA)	June 6th 2011 and Sept 26th 2012	27° 28.19 N 80° 12.76 W	14 m	Muddy sand	Anchor dredge
	5 miles station Fort Pierce (USA)	June 6th 2011 and Sept 26th 2012	27° 30.01 N 80° 12.69 W	15 m	Shell gravel	Anchor dredge
	6 miles station Fort Pierce (USA)	June 6th 2011 and Sept 26th 2012	21° 29.18 N 80° 10.98 W	15 m	“Amphioxus sand”	Anchor dredge
*E. spinifurca*	3 miles station Fort Pierce (USA)	June 27th 2011 and Sept 26th 2012 June 27th 2011 and Sept 26th 2012	27° 28.33 N 80° 13.68 W	10 m	Muddy sand muddy sand	Anchor dredge
	4 miles station Fort Pierce (USA)		27° 28.19 N 80° 12.76 W	14 m		Anchor dredge
	5 miles station Fort Pierce (USA)	June 6th 2011 and Sept 26th 2012	27° 30.01 N 80° 12.69 W	15 m	Shell gravel	Anchor dredge
	6 miles station Fort Pierce (USA)	June 6th 2011 and Sept 26th 2012	21° 29.18 N 80° 10.98 W	15 m	“Amphioxus sand”	Anchor dredge
*Echinoderes* sp.	Off shore Albufeira (Potugal)	April 28th 2012	36° 57.84 N 8° 12.63 W	45 m	Shell gravel	Higgins meiobenthic dredge/Van Veen grab

### Scanning electron microscopy (SEM)

Fixed specimens were dehydrated through a graded series of ethanol dilutions, and dried within a Tousimis Samdri-790 Critical Point Dryer (Tousimis Research Corp., Rockville, MD) with CO_2_ as an intermediate. Dried specimens were mounted on aluminum stubs, sputter coated with a gold-palladium alloy, and imaged with either a HITACHI S4800 or a JEOL JSM 6335 field emission scanning electron microscope.

### Phalloidin and propidium iodide staining

Fixed specimens of *E. spinifurca* (n = 33), *E. horni* (n = 25)*, E. dujardinii* (n = 28), *E. hispanicus* (n = 5) and *E.* sp. (n = 14) were washed and labeled in multiple species-specific experiments. For each species, specimens were washed from PBS:NaN_3_ solution with 3 × 15 min exchanges of 0.1 M PBS, followed by permeabilization in PBT (0.1 M PBS + 5.0% Triton X-100) for 24 hours at 4°C. Filamentous Actin (F-actin) fibers of musculature were labeled by incubating specimens at concentrations of a 1:40 or 1:100 dilution of Alexa Fluor® 488, 546, or 633-conjugated phalloidin (Molecular Probes) in PBT. Incubation was always performed in the dark while rocking at 4°C in glass spot plates. Total incubation times were typically 72 hrs with fresh staining solutions added after 24 and 48 hrs. For DNA staining, fixed specimens were rinsed from PBS:NaN_3_ solution with 3 × 15 min exchanges 0.1 M PBS, followed by 3 × 15 min exchanges of PBT (0.1 M PBS + 0.2% Triton X-100+ 0.5% BSA). These specimens were then treated with RNase A at 1.0 mg/ml PBT for 1.0 hr at 37°C, washed with 3 × 15 min exchanges of PBT, and incubated with propidium iodide (PI) at 5.0 μg/ml PBT in the dark while rocking for a period of 48–72 hrs at 4°C. Subsets of specimens were also co-labeled with phalloidin and propidium iodide for 48–72 hrs at 4°C. All labeling experiments were terminated with multiple exchanges of 0.1 M PBS immediately prior mounting.

### Mounting and clearing

Prior to mounting, two or three layered strips of clear tape were each placed in parallel on one side of glass microscope slide, to elevate the placement of a coverslip above the specimens. A spot of double-stick tape was placed between the clear strips. Stained specimens were placed onto the double-stick tape and oriented during adhesion. To clear the specimens, slides were transferred through graded series of isopropanol dilutions, terminating with final immersion and mounting in a 2:1 mixture of benzyl benzoate and benzyl alcohol. A coverslip was placed across the strips of clear tape and sealed with clear nail polish. Alternatively, specimens were slowly moved through a glycerol series (20%, 40%, 60%, 80%) to prevent contraction of the trunk region, and then mounted in glycerol (80% glycerol, 0.1 M PBS), or Fluoromount G® (Southern Biotech) antifade mounting medium.

### Confocal Laser Scanning Microscopy (CLSM) and 3D reconstruction

Confocal imaging was performed with an LSM 510 (Carl Zeiss Inc.), an LSM700 mounted on an Axio Imager upright microscope (Carl Zeiss Inc.), or a Leica DM 5000 CS with SP5 laser scanning unit. Confocal z-stack projections were compiled and analyzed with Fiji, v. 1.47 (Wayne Rasband, National Institutes of Health), and edited with Adobe Photoshop CS4 (Adobe Systems Incorporated, San Jose, CA). Autofluorescence of the cuticle was detectable in each of three confocal channels. Imaging of the cuticle was routinely performed with an excitation wavelength of 488 nm, and a band pass filter (BP 505–530 nm), to eliminated fluorescent emission crosstalk between adjacent channels. The cuticle, including all cuticular structures, was imaged separately, or simultaneously with the excitation of additional fluorescent markers to distinguish autofluorescent signals from the emission of fluorophore conjugates. Cuticle imaging served to orientate and guide interpretations of the internal and external anatomy in each species. For 3D reconstructions, z-stacks were surface-rendered by using the software Imaris v. 7.5.0 (Bitplane AG, Zürich, Switzerland). Schematics and figure plates were prepared with Adobe Illustrator CS6 (Adobe Systems Incorporated, San Jose, CA). The terminology used for external morphology and position follows Pardos et al. [49], Neuhaus and Higgins [14], and Sørensen and Pardos [52]; accordingly, trunk segments are numbered 1 to 11, from anterior-to-posterior. Internal structures are named in accordance with previous studies based on CLSM (see [5,29,30]) and TEM (see [1,33]). The use of Kinorhyncha in the laboratory does not raise any ethical issues and therefore Regional or Local Research Ethics Committee approvals are not required.

## Competing interests

The authors declare that they have no competing interests.

## Authors’ contributions

MH contributed in project design, field collections, CLSM imaging and analysis, designing and building the images and writing of the manuscript. MJB participated in project design, field collections, CLSM imaging and analysis, and writing of the manuscript. FP assisted in writing the manuscript, data interpretation and building images. RCN contributed with field collections, CLSM imaging and analysis, and writing the manuscript. All authors read and approved the final manuscript.

## Supplementary Material

Additional file 1 **Myoanatomy of the head of *Echinoderes spinifurca.*** Phalloidin staining and 3D reconstruction of the anterior region showing distinct muscle groups of the mouth cone, introvert and pharyngeal sheath. This specimen rotates on all three axes, beginning and ending with apical views. The introvert and mouth cone are fully extended. The pharynx is surrounded by a tulip-shaped muscular sheath (pale yellow) and is extended to reach the mouth cone (yellow). The oral styles and scalids (grayscale) do not have muscles. White lines on each side of the head correspond to the long terminal spines of this dorsoventrally curved specimen. Musculature is color-coded for the same specimen in Figure 2A-D of the manuscript. Click here for file

Additional file 2 **Myoanatomy of the body of *Echinoderes spinifurca.*** Phalloidin staining and 3D reconstruction of all muscle groups within the head, neck and trunk of the body. This specimen rotates 360° on its anterior-posterior axis, with anterior to the top. The introvert is partially everted. Segmented muscle groups of the trunk (ventral, dorsal, dorsoventral, diagonal) are visible in turquoise, blue, magenta and red, respectively. Autofluorescence of the cuticle (grayscale) shows eleven segments and spines of the trunk and terminal segment. The musculature is color-coded as in Figure 8 of the manuscript. Click here for file

Additional file 3 **Eversion, retraction and motility of *Echinoderes.*** Video of a live specimen of *E. spinifurca* under brightfield illumination. Eversion and retraction of the introvert and associated spinoscalids of the head region facilitate forward movements of the animal. The segmented trunk is highly flexible and exhibits a broad range of dorsoventral and lateral articulation. The entire gut system (mouth cone, pharynx, intestine) moves within the body as the introvert is everted and retracted. The pair of red spots at the anterior and correspond to photoreceptors. Click here for file
